# Structural-information guided fusion for spatial domain identification from spatial transcriptomics

**DOI:** 10.1093/bioinformatics/btag508

**Published:** 2026-07-09

**Authors:** Min Zhang, Peng Gao, Cheng Chen, Xiaoke Ma, Xin Chen, Shaoqing Feng

**Affiliations:** School of Computer Science and Technology, Xidian University, Xi’an, Shaanxi 710071, China; Key Laboratory of Smart Human-Computer Interaction and Wearable Technology of Shaanxi Province, Xidian University, Xi’an, Shaanxi 710071, China; Shenzhen Loop Area Institute, The First Affiliated Hospital of Xi’an jiaotong University, Xi’an, Shaanxi 710061, China; MOE Key Laboratory of Bioinformatics, BNRIST Bioinformatics Division, Institute for Precision Medicine & Department of Automation, Tsinghua University, Beijing 100084, China; School of Computer Science and Technology, Xidian University, Xi’an, Shaanxi 710071, China; Key Laboratory of Smart Human-Computer Interaction and Wearable Technology of Shaanxi Province, Xidian University, Xi’an, Shaanxi 710071, China; Fubao District, Shenzhen Loop Area Institute, Shenzhen, Guangdong 518017, China; Department of Radiology, School of Medicine, The Second Affiliated Hospital of South China University of Technology (Guangzhou First People’s Hospital), Guangzhou 510180, China; Department of Plastic and Reconstructive Surgery, Shanghai Ninth People’s Hospital, Shanghai Jiaotong University, Shanghai 200011, China

## Abstract

**Motivation:**

Accurate spatial domain identification is essential for understanding tissue organization and pathological mechanisms in spatial transcriptomics. However, existing methods mainly rely on expression profiles and spatial coordinates. Intercellular interactions are often overlooked. At the same time, preserving both local neighborhood continuity and global topological structure remains difficult.

**Results:**

We propose SGFST (*S*tructural-information *G*uided *F*usion for spatial domain identification from *S*patial *T*ranscriptomics), a novel framework for spatial domain identification in spatial transcriptomics. SGFST integrates a spatial graph and a signal graph, and employs a dual-branch graph convolutional network with attention-based fusion to capture complementary spatial and functional information. In addition, SGFST jointly optimizes a Bayesian personalized ranking loss, a zero-inflated negative binomial loss, and a distance structural information constraint to preserve local neighborhood continuity, reconstruct expression signals, and maintain global topological consistency. Experimental results on multiple datasets demonstrate that SGFST outperforms several state-of-the-art methods in spatial domain identification.

**Availability and implementation:**

The code of SGFST is available at Github (https://github.com/xkmaxidian/SGFST) and Zenodo (DOI: 10.5281/zenodo.20624899).

## 1 Introduction

Single-cell RNA sequencing (scRNA-seq) has been used to effectively trace these cell types. Specifically, scRNA-seq generates gene expression data by dissociating cells before sequencing (Andrews *et al*. 2021, Gulati *et al*. 2025). However, this process leads to the loss of the spatial coordination of cells, thereby failing to fully characterize tissue microenvironments and capturing only expression information. Fortunately, spatial transcriptomics technologies can simultaneously measure the gene expression profiles and spatial information of cells, offering new possibilities for tissue structure identification (Li *et al*. 2022, Tang *et al*. 2026).

Current spatial transcriptomics technologies can be broadly categorized into imaging-based methods and next-generation sequencing (NGS)-based methods (Williams *et al*. 2022). Imaging-based approaches mainly include fluorescence in situ hybridization (FISH) and its advanced variants, such as multiplexed error-robust in situ hybridization (MERFISH) (Chen *et al*. 2015). Although imaging-based methods provide high spatial resolution, most of them rely on predefined gene panels and therefore cannot achieve unbiased whole-transcriptome profiling. To overcome these limitations, NGS-based technologies, including Slide-seq (Rodriques *et al*. 2019), Stereo-seq (Wei *et al*. 2022), and 10× Visium (Ståhl *et al*. 2016), employ spatial barcoding strategies to enable genome-wide transcriptional profiling, thereby facilitating a more comprehensive understanding of tissue structure and function.

High-throughput gene expression and high-resolution spatial information provide the basis for spatial transcriptomics analysis. A key task in this field is to identify spatial domains with similar expression patterns (Lázár and Lundeberg 2026, Tang *et al*. 2026). However, transcriptomic data are often affected by noise and dropout, which can obscure true biological signals and lead to inaccurate domain boundaries. Therefore, robust computational methods are needed to accurately identify spatial domains from noisy spatial transcriptomics data (Yuan *et al*. 2024). Existing methods for spatial domain identification can be divided into two categories: non-spatial clustering methods and spatial clustering methods. Non-spatial methods typically rely solely on gene expression data and perform dimensionality reduction followed by clustering to identify spatial domains. However, these methods ignore spatial continuity between neighboring spots. As a result, relying solely on expression information may lead to fragmented spatial domains (Yuan *et al*. 2024, Yang *et al*. 2025). To address this limitation, a series of spatially methods have been developed to integrate spatial information with gene expression. Early methods, such as Giotto (Dries *et al*. 2021) and stLearn (Pham *et al.* 2020), improve spatial domain identification by incorporating spatial proximity or histological information. Compared with non-spatial methods, these approaches are better able to preserve spatial continuity within tissues.

Recent spatial domain identification methods have increasingly incorporated spatial neighborhood information into representation learning (Wu *et al*. 2021). SpaGCN (Hu *et al*. 2021) represents an early graph convolutional framework that integrates gene expression with spatial context. STAGATE (Dong and Zhang 2022) and DeepST (Xu *et al*. 2022) further extend this direction by using graph autoencoder-based or deep representation learning designs to obtain spatially informed embeddings. Later methods, including ST-SCSR (Zhang *et al*. 2024), DisConST (Zhen *et al*. 2026), MAEST (Zhu *et al*. 2025), SpaMask (Min *et al*. 2025), and SpaICL (Zhao and Min 2025), improve representation learning through self-representation, contrastive learning, masked graph modeling, or histology-guided learning. In parallel, BANKSY (Singhal *et al*. 2024) and MNMST (Wang *et al*. 2024) enhance spatial representation by introducing neighborhood features or multi-view information. Other related methods, such as SpaBatch (Niu and Min 2025) and mclSTExp (Min *et al*. 2024), further broaden spatial transcriptomics analysis to cross-slice integration, 3D domain identification, and histology-based expression prediction. Although these methods have substantially improved spatial transcriptomics analysis, many spatial clustering methods still depend on local neighborhood aggregation, image-derived features, or embedding-level constraints. This may lead to oversmoothed features and weaken fine-grained spatial heterogeneity, especially in complex tissues such as tumors.

While multi-view approaches like Spatial-MGCN (Wang *et al*. 2023) improve representations, most methods still overlook higher-order functional contexts such as pathway activity. Although SiDMGF (Ma *et al*. 2026) introduces pathway information, it lacks a unified objective to simultaneously coordinate local structural preservation, global topological consistency, and expression reconstruction.

**Figure 1:**
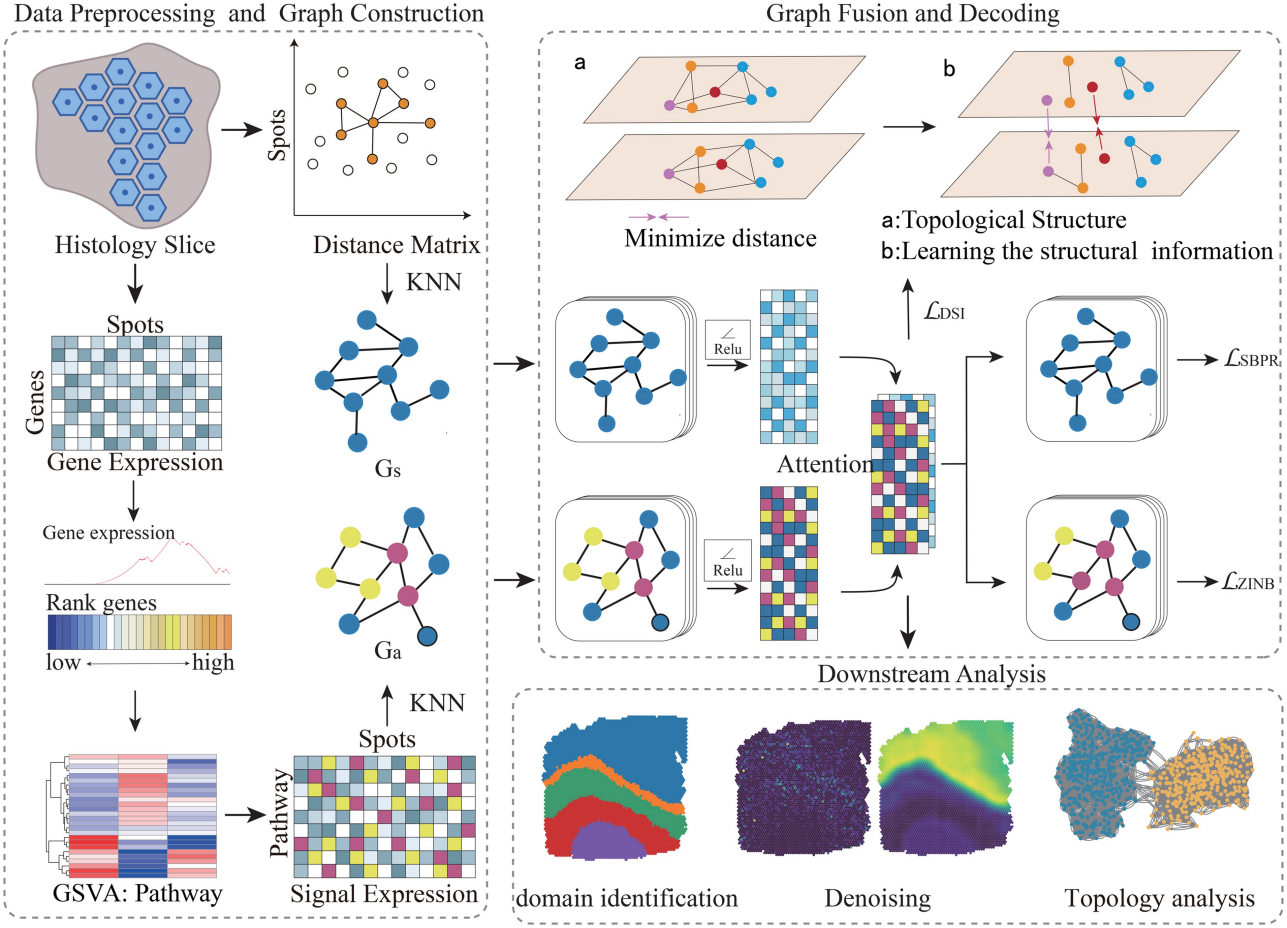
Overview of the SGFST framework. SGFST first constructs a spatial graph and a signal graph, and then learns complementary representations through a dual-branch GCN with attention fusion. The model is optimized by jointly combining structural ranking, expression reconstruction, and structural consistency constraints, and the learned embeddings are used for spatial domain identification, topology analysis, and denoising.

To address these limitations, we propose SGFST, a structural-information-guided fusion framework for spatial transcriptomics (Fig. 1). SGFST constructs a spatial graph from physical coordinates and a signal graph from inferred pathway activities to capture complementary structural and functional relationships. These views are then integrated via a dual-branch graph convolutional network with attention-based fusion. The model is jointly optimized using structural ranking, zero-inflated negative binomial (ZINB), and distance structural information (DSI) constraints, enabling it to simultaneously preserve local neighborhoods, re construct expression signals, and maintain global topological consistency. Extensive experiments confirm that SGFST achieves superior performance and provides biologically meaningful insights into tissue organization. The main contributions of this study can be summarized as follows:

- We propose SGFST, a structural-information-guided fusion framework that integrates spatial topology and pathway-level functional relationships for spatial domain identification.
- We design a unified objective that combines structural ranking, ZINB reconstruction, and DSI-based consistency, enabling SGFST to jointly preserve local neighborhoods, model noisy expression signals, and maintain global topology.
- Compared to state-of-the-art baselines, SGFST achieves superior performance in spatial domain identification and provides biologically meaningful insights into tissue organization.

## 2 Methods

### 2.1 Data preprocessing

SGFST takes spatial transcriptomics data and pathway information as inputs, where preprocessing is first performed for both. Specifically, for the spatial transcriptomics data, low-quality spots and low-abundance genes are removed. The top 3000 highly variable genes are retained for downstream analysis (Palla *et al*. 2022). We normalize the gene expression matrix by library size using SCANPY (Wolf *et al*. 2018). The normalized matrix is then log-transformed and scaled to reduce technical variation and improve comparability across spots. The ZINB reconstruction loss was computed on the non-negative normalized expression matrix before log transformation, rather than on scaled values.

Furthermore, the KEGG pathway database (Kanehisa *et al*. 2017) is employed for pathway-level analysis to introduce functional information except gene expression. GSVA is performed on the preprocessed expression matrix to estimate pathway activity scores spot (Hänzelmann *et al*. 2013), thereby obtaining a pathway activity matrix, where row *i* corresponds to the *i*-th spot and column *j* corresponds to the *j*-th pathway. Based on this matrix, a signal graph is further constructed to characterize functional similarity between spots.

### 2.2 Construction of spatial and signal graphs

Based on the spatial coordinates of the spots, we construct a spatial graph $G_{s}=(V,E_{s})$ with a corresponding binary adjacency matrix $A_{s}\in R^{n\times n}$. Specifically, we set ${\left(A_{s}\right)}_{ij}=1$ if the Euclidean distance $\|s_{i}-s_{j}{\|}_{2}$ between the coordinates of spot *i* and spot *j* is less than a predefined neighborhood radius *r*, and ${\left(A_{s}\right)}_{ij}=0$ otherwise. We construct the signal graph $G_{a}=(V,E_{a})$ and its adjacency matrix $A_{a}\in R^{n\times n}$ by evaluating the cosine similarity of pathway activities between neighboring spots. Specifically, for the pathway activity vectors $a_{i}$ and $a_{j}$ of spots *i* and *j*, we set ${\left(A_{a}\right)}_{ij}=\frac{a_{i}a_{j}^{⊤}}{\|a_{i}{\|}_{2}\|a_{j}{\|}_{2}}$ if *j* is among the *k*-nearest neighbors of *i* (i.e., $j\in N_{k}(i)$), and ${\left(A_{a}\right)}_{ij}=0$ otherwise. The spatial radius r was set according to each dataset’s coordinate scale and resolution, while k was fixed to 15 for all signal graphs.

### 2.3 Multi-graph fusion

After constructing the spatial graph and the signal graph, SGFST learns node representations from the two graphs with separate GNN encoders and then integrates them into a unified embedding. Specifically, the representation learned from the spatial graph is updated as


(1) $$H_{s}^{\left(l+1\right)}=\sigma \left({\tilde{D}}_{s}^{-\frac{1}{2}}{\tilde{G}}_{s}{\tilde{D}}_{s}^{-\frac{1}{2}}H_{s}^{\left(l\right)}W_{s}^{\left(l\right)}\right),$$

Where σ(·) denotes the activation function, $H_{s}^{\left(l\right)}$ is the node representation at the *l*-th layer, and $H_{s}^{\left(0\right)}=X$ is the input gene expression matrix. ${\tilde{G}}_{s}=G_{s}+I$ denotes the spatial adjacency matrix with self-loops, ${\tilde{D}}_{s}$ is degree matrix, and $W_{s}^{\left(l\right)}$ is the trainable weight matrix of the *l*-th layer. In the same way, the representation learned

From the signal graph is given by


(2) $$H_{a}^{\left(l+1\right)}=\sigma \left({\tilde{D}}_{a}^{-\frac{1}{2}}{\tilde{G}}_{a}{\tilde{D}}_{a}^{-\frac{1}{2}}H_{a}^{\left(l\right)}W_{a}^{\left(l\right)}\right).$$

To adaptively combine the information from the two graphs, an attention mechanism is introduced to assign node-specific weights to the spatial and signal branches. For the *i*-th node, the attention score of each branch is computed as


(3) $$e_{i}^{\left(m\right)}=W_{2}^{\left(m\right)}\tanh\left(W_{1}^{\left(m\right)}h_{i}^{\left(m\right)}+b^{\left(m\right)}\right),\;m\in \{s,a\},$$

Where $h_{i}^{\left(s\right)}$ and $h_{i}^{\left(a\right)}$ denote the embeddings of node *i* learned from the spatial graph and the signal graph, respectively, and $W_{1}^{\left(m\right)}$, $W_{2}^{\left(m\right)}$, and $b^{\left(m\right)}$ are trainable parameters. The attention weights are then normalized by a softmax function. Based on these attention weights, the final fused embedding is obtained by $H=\text{MLP}\left(e^{\left(s\right)}⊙H_{s}+e^{\left(a\right)}⊙H_{a}\right),$ where ⊙ denotes element-wise multiplication. In this way, the fused representation can adaptively integrate spatial structural information and pathway-related functional information, providing a comprehensive embedding for downstream analysis.

### 2.4 BPR-based structural ranking loss

To preserve the structural information of the graph, we employ a Bayesian Personalized Ranking (BPR) loss for structural supervision (Rendle *et al*. 2012). The structure decoder takes the fused embedding *H* as input and outputs an edge score matrix ${\tilde{G}}_{s}\in R^{n\times n}$. Here, ${\tilde{G}}_{s,ij}$ denotes the predicted score of the connection between nodes *i* and *j*. At each epoch, up to 4,096 positive edges were sampled from the undirected spatial graph, with three non-neighboring negative spots sampled for each positive edge. Self-pairs and observed spatial neighbors were excluded, and negative samples were re-sampled online during training. The same number of negative samples is used for each anchor spot to ensure balanced structural supervision. That is, ${\tilde{G}}_{s,ij}>{\tilde{G}}_{s,ik}.$ Based on this idea, the structural ranking loss is defined as


(4) $$L_{\text{str}}=\frac{1}{\left|\Omega \right|}\sum_{(i,j,k)\in \Omega }\;\text{log}\;\left(1+\text{exp}\;\left(-({\tilde{G}}_{s,ij}-{\tilde{G}}_{s,ik})\right)\right),$$

Where Ω denotes the set of sampled triplets. By prioritizing higher scores for connected over disconnected node pairs, this loss optimizes relative rankings. Given the inherent sparsity of spatial graphs, this ranking-based objective is significantly more robust than absolute reconstruction for preserving local neighborhood consistency.

### 2.5 Expression reconstruction with ZINB modeling

To reconstruct gene expression from the fused representation, we model gene expression counts in a probabilistic model. Since spatial transcriptomics data are often over-dispersed and contain excessive zeros, we use a zero-inflated negative binomial (ZINB) model for reconstruction. Let $x_{i}$ denote the observed count of gene *g* at spot *i*. We first describe $x_{i}$ with a negative binomial (NB) distribution (Hafemeister and Satija 2019):


$$\text{NB}(x_{i}|\mu_{i},\theta_{i})=\frac{\Gamma (x_{i}+\theta_{i})}{x_{i}!\Gamma (\theta_{i})}{\left(\frac{\theta_{i}}{\mu_{i}+\theta_{i}}\right)}^{\theta_{i}}{\left(\frac{\mu_{i}}{\mu_{i}+\theta_{i}}\right)}^{x_{i}},$$

Where $\mu_{i}$ and $\theta_{i}$ denote the mean and dispersion parameters, respectively. To further account for the excessive zeros caused by technical dropout, we introduce a zero-inflation parameter $\pi_{i}$, which leads to the ZINB model:


$$\text{ZINB}(x_{i}|\pi_{i},\mu_{i},\theta_{i})=\pi_{i}1(x_{i}=0)+(1-\pi_{i})\text{NB}(x_{i}|\mu_{i},\theta_{i}),$$

Where $\pi_{i}$ represents the probability that the observation belongs to the extra zero-inflation component, and 1(·) is the indicator function. We define the expression reconstruction loss as the negative log-likelihood of the observed expression counts under the ZINB distribution.


$$L_{\text{ZINB}}=-\sum_{i=1}^{n}\sum_{g=1}^{p}\;\text{log}\;\text{ZINB}(x_{i}|\pi_{i},\mu_{i},\theta_{i}),$$

Where *n* and *p* denote the numbers of spots and genes, respectively. In practice, the expression decoder takes the fused representation as input and predicts the three ZINB parameters, π, μ, and θ. We then fit the observed expression matrix by minimizing the ZINB negative log-likelihood loss.

### 2.6 Structural information consistency

To maintain global consistency with prior graph structures, a structure-consistency constraint based on graph entropy is introduced. Specifically, a shared graph $C=P(HH^{⊤})\in R^{n\times n}$ is constructed, where *H* is the fused embedding, and P(·) is a post-processing operator that applies symmetrization, non-negativity enforcement, and diagonal removal to the pairwise similarity matrix. To characterize the global topological structure of a graph, we employ graph entropy to quantify the distribution of edge weights. For a graph *G* with adjacency matrix *A*, its graph entropy is defined as


(5) $$H(G)=-\sum_{i,j}\frac{A_{ij}}{\text{vol}(A)}\;\text{log}\;\left(\frac{A_{ij}}{\text{vol}(A)}\right),$$

Where $A_{ij}$ represents the edge weight between the *i*-th and *j*-th vertices in graph *G*, and $\text{vol}(A)=\sum_{i,j}A_{ij}$ denotes the volume of graph *G*. To measure the structural similarity between the shared graph *C* and the reference graph $G_{s}$, we further construct their average distribution matrix *M*, whose entries are defined as $m_{ij}=\frac{1}{2}\left(\frac{C_{ij}}{\text{vol}(C)}+\frac{{\left(G_{s}\right)}_{ij}}{\text{vol}(G_{s})}\right).$ If the shared graph *C* and the spatial graph $G_{s}$ have similar global edge-weight distributions, the graph entropy of the average matrix *M* should be close to the mean of H(C) and $H(G_{s})$. Otherwise, the deviation becomes larger when their structures differ significantly. Based on this intuition, we define the distance structural information (DSI) between *C* and $G_{s}$ as


(6) $$D_{\text{SI}}(C,G_{s})=\left|H(M)-\frac{1}{2}(H(C)+H(G_{s}))\right|.$$

Similarly, the structural discrepancy between the shared graph *C* and the signal graph $G_{a}$ is defined as $D_{\text{SI}}(C,G_{a})$. To preserve global structural consistency, we encourage the shared graph *C* to align with both the spatial graph $G_{s}$ and the signal graph $G_{a}$. The resulting global structure-consistency loss is defined a


(7) $$L_{\text{DSI}}=D_{\text{SI}}(C,G_{s})+D_{\text{SI}}(C,G_{a}).$$

This constraint does not enforce element-wise similarity between the shared graph and the prior graphs. Instead, it encourages consistency in their overall edge-weight distributions. As a result, the model can better preserve global structural agreement across different graph views. The overall objective function is defined as


(8) $$L=\lambda_{1}L_{\text{SBPR}}+\lambda_{2}L_{\text{ZINB}}+\lambda_{3}L_{\text{DSI}}.$$

Where $\lambda_{1}$, $\lambda_{2}$, and $\lambda_{3}$ are hyperparameters that control the contributions of structural ranking, expression reconstruction, and global structure consistency, respectively.

### 2.7 Clustering and visualizationg

SGFST first uses a GCN to learn low-dimensional node embeddings, and then applies K-means to these embeddings to identify spatial domains. The number of clusters can be preset according to prior knowledge. For visualization, the learned embeddings are further projected into a low-dimensional space using Uniform Manifold Approximation and Projection (UMAP), which provides an intuitive view of the identified spatial domains.

### 2.8 Baselines and criteria

We selected the most representative spatial domain identification methods as baseline approaches, including SCANPY (Wolf *et al*. 2018), Giotto (Dries *et al*. 2021), stLearn(Pham *et al.* 2020), SpaGCN (Hu *et al*. 2021), DisConST (Zhen *et al*. 2026), STAGATE (Dong and Zhang 2022), BANKSY (Singhal *et al*. 2024), MNMST (Wang *et al*. 2024), DeepST (Xu *et al*. 2022), ST-SCSR (Zhang *et al*. 2024), Spatial-MGCN (Wang *et al*. 2023), MuCST (Wang *et al*. 2025), MAEST (Zhu *et al*. 2025) and SiDMGF (Ma *et al*. 2026).

### 2.9 Computational complexity

Let *n* denote the number of spots/cells, *d* the embedding dimension, and $\left|E_{s}\right|$ and $\left|E_{a}\right|$ the numbers of edges in the spatial and signal graphs. The GCN encoding step scales as $O((|E_{s}|+|E_{a}|)d)$ per layer with sparse graph operations. The BPR loss is computed on a fixed number of sampled triplets, with at most 4,096 positive edges and three negative samples per positive edge at each epoch. The main computational bottleneck is the shared graph construction $C=P(HH^{⊤})$, which requires $O(n^{2}d)$ time and $O(n^{2})$ memory. SGFST is currently trained in a full-batch manner; for larger datasets, top-*k* sparse similarity or block-wise computation can be used to reduce the cost.

To quantify performance of various algorithms, the adjusted Rand Index (ARI) (Hubert and Arabie 1985) is selected as measurements.

## 3 Result

### 3.1 Overview of the SGFST workflow

The ultimate goal of SGFST is to accurately identify spatial domains by effectively integrating spatial topology, pathway-related functional information, and gene expression signals within a unified framework. As shown in Fig. 1, SGFST fulfills this goal through four procedures: data preprocessing, graph construction, graph fusion and decoding, and downstream analysis. First, the input spatial transcriptomics data are preprocessed, and pathway activity profiles are inferred from gene expression data to introduce functional information beyond gene-level expression. Second, based on spot coordinates and pathway activity profiles, SGFST constructs a spatial graph and a signal graph to characterize complementary structural and functional relationships among spots. Third, SGFST performs graph fusion and decoding to integrate the two graphs within a unified framework. Specifically, the model combines structural ranking, ZINB-based expression reconstruction, and DSI-based structural consistency to preserve local neighborhood structure, recover expression signals, and maintain global topological consistency, respectively. In this way, SGFST jointly preserves local neighborhood structure, maintains global topological consistency, and reconstructs gene expression signals under a ZINB-based probabilistic model. Finally, the learned outputs of SGFST are used for downstream analyses, including spatial domain identification, topology analysis, and denoised expression reconstruction.

### 3.2 Parameter analysis of SGFST

SGFST includes three key hyperparameters, $\lambda_{1}$, $\lambda_{2}$, and $\lambda_{3}$, which respectively control the contributions of the structural ranking loss, the ZINB modeling loss, and the structural information consistency loss. The effects of these parameters on the performance of SGFST on the DLPFC dataset (Maynard *et al*. 2021) were used pairwise grid search (Fig. S1, available as supplementary data at *Bioinformatics* online). The results show that SGFST achieves consistently strong performance when $\lambda_{1}\in [0.1,100]$, $\lambda_{2}\in [0.001,0.1]$, and $\lambda_{3}\in [0.001,100]$, indicating that the model is relatively robust over broad parameter ranges. There are some good reasons to explain this tendency. If $\lambda_{1}$ is too small, local neighborhood supervision becomes insufficient to guide the embedding. For $\lambda_{2}$, a small value severely weakens expression reconstruction, reducing the ability of model to handle dropout and dispersion. In contrast, the primary risk for $\lambda_{3}$ lies in being excessively large, which may over-constrain the model and sacrifice its flexibility in capturing fine-grained spatial heterogeneity. Overall, these results demonstrate that SGFST remains stable across relatively wide parameter ranges. In all experiments, we set $\lambda_{1}=1$, $\lambda_{2}=0.1$, and $\lambda_{3}=0.01$.

### 3.3 SGFST accurately identifies spatial domains in various ST datasets with the 10× Visium platform

On the 10× Visium platform, we first evaluated the performance of multiple algorithms on the dorsolateral prefrontal cortex (DLPFC) dataset. The dataset consists of 12 slices obtained from three human brains. Based on known marker genes and morphological features, each slice was annotated into six cortical layers (Layer 1 to Layer 6) and the white matter layer, and thus serves as a benchmark dataset for spatial domain identification. A total of 14 state-of-the-art methods were selected as baselines to validate the performance of the proposed method (see the Materials and Methods section).

**Figure 2:**
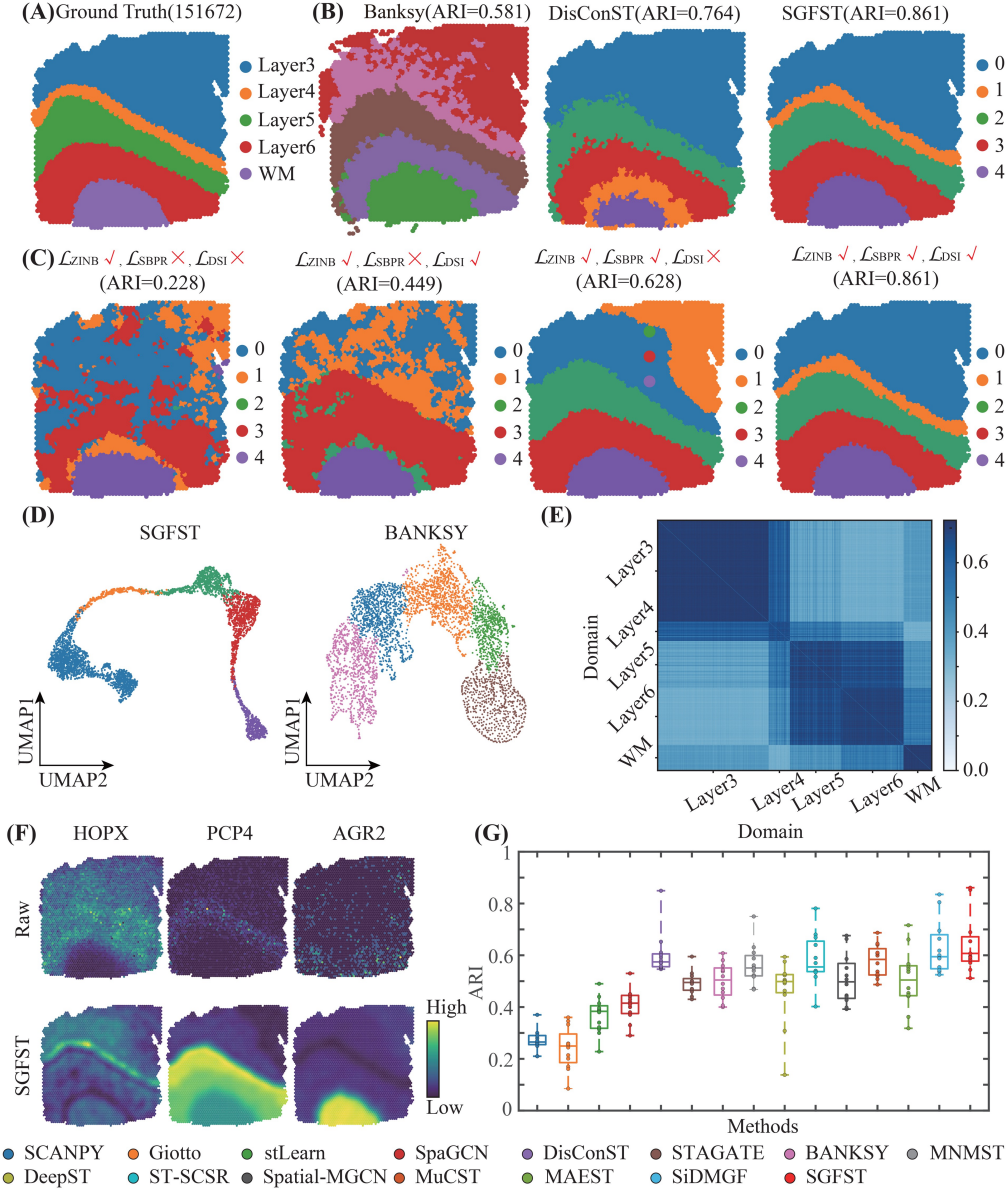
SGFST accurately identifies spatial domains in the DLPFC dataset. (A) Ground-truth spatial domains of slice 151672. (B) Spatial domain identification results of SGFST and representative baseline methods. (C) Ablation study of different loss terms in SGFST. (D) UMAP visualization of the learned embeddings by SGFST and BANKSY. (E) Domain correspondence matrix between identified domains and annotated cortical layers. (F) Spatial expression patterns of representative genes in the raw and reconstructed expression profiles. (G) ARI comparison of all methods across 12 DLPFC slices.

Figure 2A illustrates the ground-truth spatial domains of slice 151 672 from the DLPFC dataset, where each domain is represented by a distinct color. We applied all baseline methods to this slice for comparison. As shown in Fig. 2B and Supplementary Figs. S2–S4, available as supplementary data at *Bioinformatics* online, SGFST achieved the closest spatial domain pattern to the ground truth, demonstrating its superior ability to characterize the spatial organization of brain tissue. Specifically, SGFST achieved an ARI of 0.861, while BANKSY and DisConST obtained ARI values of 0.581 and 0.764, respectively. In addition, we have added the full ablation results for all parameter combinations on this dataset to the Supplementary Material.

These methods still have limited ability to explicitly coordinate local neighborhood preservation with global topological consistency. In contrast, SGFST integrates complementary structural information at multiple levels and uses attention-based fusion to adaptively balance spatial and functional signals. As a result, SGFST learns more discriminative embeddings and achieves the highest ARI on these slices.

To further elucidate the source of the performance gain of SGFST, we conducted an ablation study by progressively removing its three objective terms (Fig. 2C). Using only the ZINB reconstruction term resulted in fragmented spatial domains (ARI = 0.228). Incorporating the structural information consistency term increased the ARI to 0.449, helping preserve the overall tissue organization. Further introducing the structural ranking loss raised the ARI to 0.628 by maintaining local neighborhood continuity. The full model achieved the best performance (ARI = 0.861), confirming the complementary contributions of all three terms. Furthermore, UMAP visualizations (Fig. 2D) showed that SGFST exhibited clearer cluster separation and a more orderly arrangement of cortical domains than BANKSY, indicating that the fused embeddings from our dual-branch encoder are highly discriminative. Finally, the domain correspondence matrix (Fig. 2E) displayed a clear block-wise pattern, confirming that the identified domains are highly consistent with annotated cortical layers.

We further assessed whether the reconstructed expression profiles retained biologically meaningful spatial structure. As shown in Fig. 2F, three differentially expressed genes, HOPX, PCP4, and AGR2, were selected for visualization. Compared with the raw expression, the reconstructed expression exhibited clearer layer structure. Finally, we further compared the ARI values of all baseline methods across the 12 DLPFC slices (Fig. 2G, Figs. S2, S3, S4, S5, available as supplementary data at *Bioinformatics* online). The results showed that SGFST consistently achieved the best performance among all compared methods, indicating that its advantage was maintained across multiple sections. These results indicate that the ZINB module not only reduces noise, but also preserves domain-relevant signals that facilitate spatial domain identification. As shown in Fig. S6, available as supplementary data at *Bioinformatics* online, SGFST achieved superior clustering performance while maintaining a moderate computational cost in terms of running time and memory usage compared with baseline methods.

We next evaluated SGFST on a 10× Visium mouse posterior brain slice featuring complex spatial architectures. Using the Allen Mouse Brain Atlas as an anatomical reference (Fig. S7A, available as supplementary data at *Bioinformatics* online), SGFST demonstrated superior agreement over baseline methods (Fig. S7B, available as supplementary data at *Bioinformatics* online). While several baselines identified major macroscopic regions like the cerebellum (CB), their segmentations were often heavily fragmented by noisy assignments. In contrast, SGFST uniquely delineated the olfactory region (OLF) and hippocampal formation (HPF) as continuous, well-preserved domains, confirming its robustness in resolving intricate tissue organization.

### 3.4 SGFST can accurately identify cancer domains in the human breast cancer spatial transcriptomics dataset

**Figure 3:**
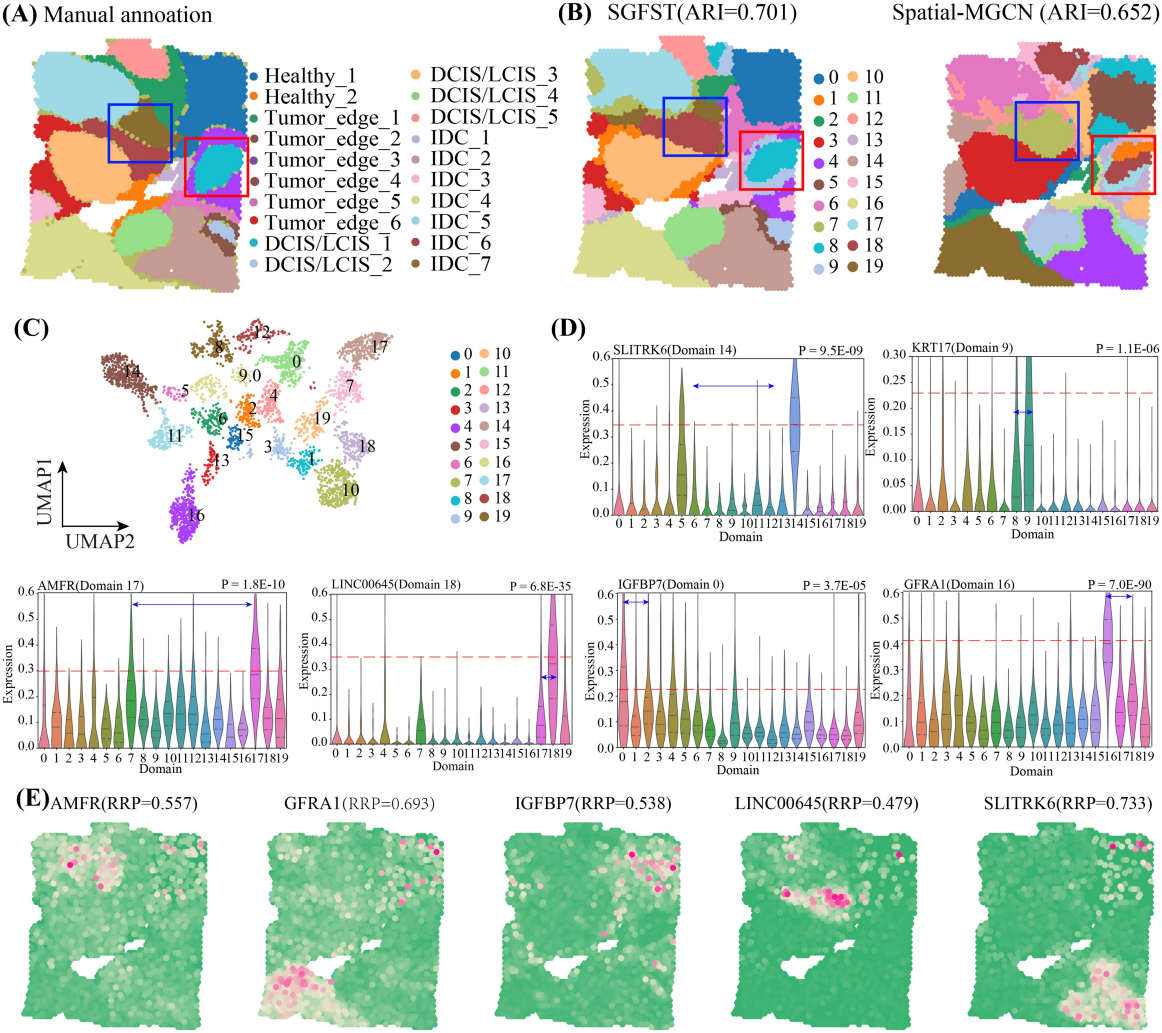
SGFST accurately identifies spatial domains in the human breast cancer dataset. (A) Manual annotation of the breast cancer tissue. (B) Comparison of spatial domain identification results produced by SGFST and Spatial-MGCN. (C) UMAP visualization of the learned embeddings. (D) Violin plots of representative marker genes across spatial domains. (E) Spatial expression maps of representative marker genes.

To examine whether SGFST can effectively identify spatial domains in tumor tissues, we applied it to the breast cancer dataset. Fig. 3A is the visualization of H&E breast cancer tissue, consisting of ductal carcinoma in situ/lobular carcinoma in situ (DCIS/LCIS), healthy region (Healthy), invasive ductal carcinoma (IDC), and tumor surrounding regions (Tumor edge). The ability to distinguish these regions was used to evaluate the capability of the learned features. For visualization, the number of domains was set to 20, consistent with the manual annotation. Under this setting, SGFST achieved clearly better performance than the competing baseline methods (Fig. S8, S12, available as supplementary data at *Bioinformatics* online). Specifically, SGFST and Spatial-MGCN achieved ARI scores of 0.701 and 0.652, respectively, while the scores of BANKSY, STAGATE, DeepST, PROST, SEDR, stLearn, SCANPY, and SpaGCN were 0.572, 0.510, 0.594, 0.574, 0.517, 0.592, 0.516, and 0.563, respectively. Moreover, Spatial-MGCN showed a tendency toward over-segmentation at domain boundaries (red region in Fig. 3B) and occasionally merged two distinct domains into a single region (blue region in Fig. 3B).

Figure S9A, available as supplementary data at *Bioinformatics* online shows the dotplot of representative marker genes across the identified domains. Several genes displayed strong domain-specific expression, suggesting that the domains detected by SGFST captured substantial transcriptional heterogeneity within the breast cancer tissue. In addition, we aim to characterize spatial domains from the perspective of topological structure. As shown in the pairwise correlation heatmap (Fig. 3B), hierarchical clustering reveals distinct modular relationships among the domains, reflecting their structured anatomical organization. In Fig. 3C, the UMAP projection of the learned embeddings shows that most domains can be clearly separated in the latent space, demonstrating the effectiveness of SGFST in learning discriminative representations. To further validate the biological relevance of the identified spatial domains, we also examine marker genes for each domain, which are significantly enriched in their corresponding regions. Violin plots (Fig. 3D) confirmed that genes such as SLITRK6, KRT17, AMFR, LINC00645, IGFBP7 and GFRA1 are significantly enriched in specific spatial domains (Student’s t-test). These genes are closely associated with the development and pathological characteristics of breast cancer. For example, AMFR promotes tumor cell motility and invasion by regulating intracellular signaling pathways, thereby accelerating cancer dissemination. IGFBP7 plays an important role in regulating cell adhesion, senescence, and angiogenesis. GFRA1, a receptor involved in neurotrophic signaling pathways, has been shown to be associated with cancer cell survival and metastasis. Overall, the domain-specific enrichment of these genes reveals substantial transcriptional heterogeneity within breast cancer tissues, suggesting distinct functional states related to proliferation, invasion, or tumor–microenvironment interactions. Pearson correlation analysis between raw and reconstructed marker-gene profiles further showed that SGFST preserved the original expression signals while improving spatial continuity, consistent with the localized patterns shown in Fig. 3E.

### 3.5 SGFST is applicable to datasets from various pltaforms

**Figure 4:**
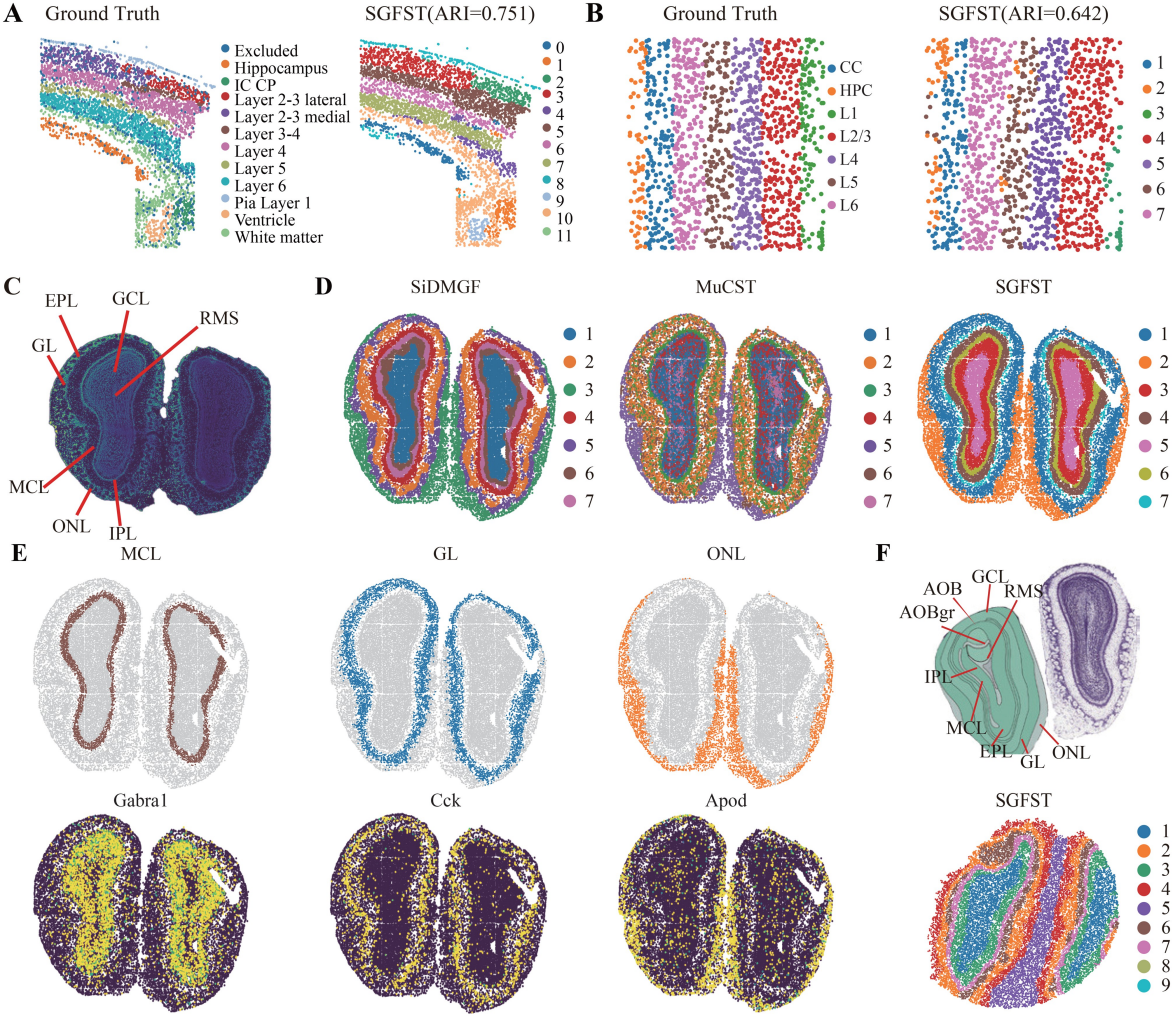
SGFST is applicable to spatial transcriptomics datasets generated by diverse platforms with different resolutions and gene coverages. (A) The manual annotations of mouse cortex dataset generated by osmFISH (left), and spatial domains identified by SGFST (right). (B) The manual annotation of mouse visual cortex sequenced by STARmap (left), and spatial domains identified by SGFST (right). (C) DAPI-stained image of mouse olfactory bulb dataset generated by Stereo-seq platform. (D) The spatial domains identified by SiDMGF, MuCST, and SGFST. (E) Visualization of spatial domains identified by SGFST, and the spatial expression patterns of known markers. (F) The mouse olfactory bulb dataset generated by the Slide-seq platform.

In addition to the 10x Visium platform, it is essential to further evaluate the preference of SGFST across datasets generated from various platforms. We first applied SGFST to the non-lattice mouse somatosensory cortex dataset generated by the osmFISH platform (Codeluppi *et al*. 2018, Wang *et al*. 2018). As shown in Fig. 4A (right), SGFST produces clear and well-defined spatial domain structures. Due to space constraints, the results of other competing methods are presented in the Supplementary Materials (Fig. S10, available as supplementary data at *Bioinformatics* online), where SGFST consistently demonstrates superior performance. Quantitatively, SGFST achieves the highest ARI score of 0.751, substantially out performing competing approaches, including BANKSY (0.338), stLearn (0.161), SCANPY (0.105), STAGATE (0.480), SpaGCN (0.443), DeepST (0.494), PROST (0.611), MNMST (0.619), ST-SCSR (0.675), Spatial-MGCN (0.271), MuCST (0.507) and SiDMGF (0.680). The results show that, compared with other methods, SGFST better identifies the complete Layer 2/3 lateral region and produces clearer boundaries.

We further evaluated all methods on the mouse visual cortex dataset generated by Starmap, which contains 1020 genes and 1207 cells, and is manually annotated into seven cortical layers (Fig. 4B, left). The results show that SGFST achieves the highest ARI among all methods. Compared with the best baseline, SiDMGF (0.601), SGFST (0.619) improves ARI by about 0.018. In addition, SGFST identifies spatial domains more clearly, with sharper boundaries and fewer ambiguous regions (Fig. 9). To investigate why SGFST precisely captures inter-layer boundaries, we visualized the reconstructed features using UMAP. The results show that SGFST exhibits strong intra-cluster compactness and clear inter-cluster separation, which baseline methods fail to achieve. These distinct clustering patterns confirm that SGFST successfully preserves both local topological relationships and a stable global structure.

We further evaluated SGFST on mouse olfactory bulb datasets generated by Stereo-seq (Xu *et al*. 2024) and Slide-seq (Stickels *et al*. 2021) (Fig. 4C and F). For the Stereo-seq data, SGFST identified spatial domains highly consistent with anatomical annotations (Fig. 4D). In contrast, MuCST yielded scattered outliers, and SiDMGF produced spatially discontinuous regions, highlighting that SGFST effectively preserves domain integrity by leveraging entropy-based structural information. This biological relevance was corroborated by spatial expression patterns, with Gabra1, Cck, and Apod tightly mapping to the identified MCL, GL, and ONL regions, respectively. Furthermore, SGFST accurately delineated complex structures like the ONL and AOB regions in the independent Slide-seq dataset. Collectively, these findings demonstrate the robustness of SGFST across platforms with varying spatial resolutions and gene coverages.

## 4 Conclusion

Accurately identifying spatial domains remains challenging due to the difficulty of simultaneously preserving local neighborhood continuity, global topological organization, and expression fidelity in noisy transcriptomics data. In this study, we addressed this problem by developing a structure-informed dual-branch framework, termed SGFST. Different from existing methods, SGFST jointly incorporates spatial topology and pathway-related functional information within a unified graph-learning model. In particular, SGFST combines a structural ranking strategy to preserve local neighborhood relationships, a ZINB-based decoder to recover noisy expression signals, and a DSI-based constraint to maintain global topological consistency. Experiments on multiple datasets showed that SGFST consistently achieved strong performance across different tissues and platforms, indicating that multi-level structural modeling is beneficial for accurate and robust spatial domain identification. Moreover, the identified domains showed good agreement with manual annotations and marker-gene expression patterns, suggesting that SGFST also provides biologically interpretable results.

There are still several opportunities to further improve SGFST in future work. First, the current framework focuses on unsupervised spatial domain identification, while the relationships among inferred domains are modeled only implicitly through the learned topology. It would be interesting to explicitly characterize inter-domain organization and hierarchical structure for downstream biological analysis. Second, although the signal graph introduces functional information beyond gene expression alone, it is still derived from predefined pathway knowledge. How to incorporate more adaptive biological priors, such as gene regulation or cell–cell communication information, remains a promising direction. Third, the current model does not integrate histological image information, which may provide complementary cues for delineating complex domain boundaries.

## Supplementary Material

btag508_Supplementary_Data

## Data Availability

All datasets used in this study are publicly available. Specifically, (1) the 12 human DLPFC slices are available from SpatialLIBD at http://spatial.libd.org/spatialLIBD; (2) the mouse posterior brain slice was generated by 10x Visium and is available from https://www.10xgenomics.com/resources/datasets; (3) the human breast cancer and DCIS slices were generated by 10× Visium and are available from https://www.10xgenomics.com/resources/datasets; (4) the mouse visual cortex slice was generated by STARmap and is available at https://figshare.com/articles/dataset/STARmap_datasets/22565209; (5) the mouse cortex somatosensory slice was generated by osmFISH and is available at http://linnarssonlab.org/osmFISH/availability; (6) the mouse olfactory bulb slice was generated by Stereo-seq and is available at https://github.com/JinmiaoChenLab/SEDR_analyses; and (7) the Slide-seqV2 dataset is available from the Broad Institute Single Cell Portal at https://singlecell.broadinstitute.org/single-cell/study/SCP815.

## References

[btag508-B1] Andrews N , Serviss JT, Geyer N et al An unsupervised method for physical cell interaction profiling of complex tissues. Nat Methods 2021;18:912–20. 34253926 10.1038/s41592-021-01196-2

[btag508-B2] Chen KH , Boettiger AN, Moffitt JR et al Spatially resolved, highly multiplexed RNA profiling in single cells. Science 2015;348:aaa6090. 25858977 10.1126/science.aaa6090 PMC4662681

[btag508-B3] Codeluppi S , Borm LE, Zeisel A et al Spatial organization of the somatosensory cortex revealed by osmfish. Nat Methods 2018;15:932–5. 30377364 10.1038/s41592-018-0175-z

[btag508-B4] Dong K , Zhang S. Deciphering spatial domains from spatially resolved transcriptomics with an adaptive graph attention auto-encoder. Nat Commun 2022;13:1739. 35365632 10.1038/s41467-022-29439-6 PMC8976049

[btag508-B5] Dries R , Zhu Q, Dong R et al Giotto: a toolbox for integrative analysis and visualization of spatial expression data. Genome Biol 2021;22:78. 33685491 10.1186/s13059-021-02286-2 PMC7938609

[btag508-B6] Gulati GS , D’Silva JP, Liu Y et al Profiling cell identity and tissue architecture with single-cell and spatial transcriptomics. Nat Rev Mol Cell Biol 2025;26:11–31. 39169166 10.1038/s41580-024-00768-2

[btag508-B7] Hafemeister C , Satija R. Normalization and variance stabilization of single-cell RNA-seq data using regularized negative binomial regression. Genome Biol 2019;20:296. 31870423 10.1186/s13059-019-1874-1 PMC6927181

[btag508-B8] Hänzelmann S , Castelo R, Guinney J. GSVA: gene set variation analysis for microarray and RNA-seq data. BMC Bioinformatics 2013;14:7. 23323831 10.1186/1471-2105-14-7 PMC3618321

[btag508-B9] Hu J , Li X, Coleman K et al SpaGCN: integrating gene expression, spatial location and histology to identify spatial domains and spatially variable genes by graph convolutional network. Nat Methods 2021;18:1342–51. 34711970 10.1038/s41592-021-01255-8

[btag508-B10] Hubert L , Arabie P. Comparing partitions. J Classif 1985;2:193–218.

[btag508-B11] Kanehisa M , Furumichi M, Tanabe M et al KEGG: new perspectives on genomes, pathways, diseases and drugs. Nucleic Acids Research 2017;45:D353–61. 27899662 10.1093/nar/gkw1092 PMC5210567

[btag508-B12] Lázár E , Lundeberg J. Spatial architecture of development and disease. Nat Rev Genet 2026;27:118–36. 41028908 10.1038/s41576-025-00892-5

[btag508-B13] Li B , Zhang W, Guo C et al Benchmarking spatial and single-cell transcriptomics integration methods for transcript distribution prediction and cell type deconvolution. Nat Methods 2022;19:662–70. 35577954 10.1038/s41592-022-01480-9

[btag508-B14] Ma Y , Wang Y, Ma X. Signal-based spatial domain identification of spatially resolved transcriptomics with multigraph fusion. Brief Bioinform 2026;27:bbag052. 41671348 10.1093/bib/bbag052 PMC12893220

[btag508-B15] Maynard KR , Collado-Torres L, Weber LM et al Transcriptome-scale spatial gene expression in the human dorsolateral prefrontal cortex. Nat Neurosci 2021;24:425–36. 33558695 10.1038/s41593-020-00787-0 PMC8095368

[btag508-B16] Min W , Shi Z, Zhang J et al Multimodal contrastive learning for spatial gene expression prediction using histology images. Brief Bioinform 2024;25:bbae551. 39471412 10.1093/bib/bbae551 PMC11952928

[btag508-B17] Min W , Fang D, Chen J et al Spamask: dual masking graph autoencoder with contrastive learning for spatial transcriptomics. PLOS Computat Biol 2025;21:e1012881. 10.1371/journal.pcbi.1012881 PMC11968113 40179332

[btag508-B18] Niu J , Min W. Spabatch: batch alignment of spatial transcriptomics data using graph deep learning. *bioRxiv* 2025:2025–03.

[btag508-B19] Palla G , Spitzer H, Klein M et al Squidpy: a scalable framework for spatial omics analysis. Nat Methods 2022;19:171–8. 35102346 10.1038/s41592-021-01358-2 PMC8828470

[btag508-B20] Pham D , Tan X, Xu J et al stlearn: integrating spatial location, tissue morphology and gene expression to find cell types, cell-cell interactions and spatial trajectories within undissociated tissues. *Biorxiv* 2020. 2020–05.

[btag508-B21] Rendle S , Freudenthaler C, Gantner Z et al BPR: Bayesian personalized ranking from implicit feedback. In: *Proceedings of the Twenty-Fifth Conference on Uncertainty in Artificial Intelligence*. 2009:452–461.

[btag508-B22] Rodriques SG , Stickels RR, Goeva A et al Slide-seq: a scalable technology for measuring genome-wide expression at high spatial resolution. Science 2019;363:1463–7. 30923225 10.1126/science.aaw1219 PMC6927209

[btag508-B23] Singhal V , Chou N, Lee J et al Banksy unifies cell typing and tissue domain segmentation for scalable spatial omics data analysis. Nat Genet 2024;56:431–41. 38413725 10.1038/s41588-024-01664-3 PMC10937399

[btag508-B24] Ståhl PL , Salmén F, Vickovic S et al Visualization and analysis of gene expression in tissue sections by spatial transcriptomics. Science 2016;353:78–82. 27365449 10.1126/science.aaf2403

[btag508-B25] Stickels RR , Murray E, Kumar P et al Highly sensitive spatial transcriptomics at near-cellular resolution with Slide-seqV2. Nat Biotechnol 2021;39:313–9. 33288904 10.1038/s41587-020-0739-1 PMC8606189

[btag508-B26] Tang J , Chen Z, Qian K et al The interpretable multimodal dimension reduction framework spahdmap enhances resolution in spatial transcriptomics. Nat Cell Biol 2026;28:363–77. 41495202 10.1038/s41556-025-01838-z PMC12904794

[btag508-B27] Wang B , Luo J, Liu Y et al Spatial-MGCN: a novel multi-view graph convolutional network for identifying spatial domains with attention mechanism. Brief Bioinform 2023;24:bbad262. 37466210 10.1093/bib/bbad262

[btag508-B28] Wang X , Allen WE, Wright MA et al Three-dimensional intact-tissue sequencing of single-cell transcriptional states. Science 2018;361:eaat5691. 29930089 10.1126/science.aat5691 PMC6339868

[btag508-B29] Wang Y , Liu Z, Ma X. Mnmst: topology of cell networks leverages identification of spatial domains from spatial transcriptomics data. Genome Biol 2024;25:133. 38783355 10.1186/s13059-024-03272-0 PMC11112797

[btag508-B30] Wang Y , Liu Z, Ma X. Mucst: restoring and integrating heterogeneous morphology images and spatial transcriptomics data with contrastive learning. Genome Med 2025;17:21. 40082941 10.1186/s13073-025-01449-1 PMC11907906

[btag508-B31] Wei X. , Fu S., Li H. et al Single-cell stereo-seq reveals induced progenitor cells involved in axolotl brain regeneration. Science 2022;377:eabp9444. 36048929 10.1126/science.abp9444

[btag508-B32] Williams CG , Lee HJ, Asatsuma T et al An introduction to spatial transcriptomics for biomedical research. Genome Med 2022;14:68. 35761361 10.1186/s13073-022-01075-1 PMC9238181

[btag508-B33] Wolf FA , Angerer P, Theis FJ. Scanpy: large-scale single-cell gene expression data analysis. Genome Biol 2018;19:15. 29409532 10.1186/s13059-017-1382-0 PMC5802054

[btag508-B34] Wu Z , Pan S, Chen F et al A comprehensive survey on graph neural networks. IEEE Trans Neural Netw Learn Syst 2020;32:4–24. 10.1109/TNNLS.2020.2978386 32217482

[btag508-B35] Xu C , Jin X, Wei S et al DeepST: identifying spatial domains in spatial transcriptomics by deep learning. Nucleic Acids Research 2022;50: e131. 36250636 10.1093/nar/gkac901 PMC9825193

[btag508-B36] Xu H , Fu H, Long Y et al Unsupervised spatially embedded deep representation of spatial transcriptomics. Genome Med 2024;16:12. 38217035 10.1186/s13073-024-01283-x PMC10790257

[btag508-B37] Yang Y , Cui Y, Zeng X et al STAIG: spatial transcriptomics analysis via image-aided graph contrastive learning for domain exploration and alignment-free integration. Nat Commun 2025;16:1067. 39870633 10.1038/s41467-025-56276-0 PMC11772580

[btag508-B38] Yuan Z , Zhao F, Lin S et al Benchmarking spatial clustering methods with spatially resolved transcriptomics data. Nat Methods 2024;21:712–22. 38491270 10.1038/s41592-024-02215-8

[btag508-B39] Zhang M , Zhang W, Ma X. ST-SCSR: identifying spatial domains in spatial transcriptomics data via structure correlation and self-representation. Brief Bioinform 2024;25:bbae437. 39228303 10.1093/bib/bbae437 PMC11372132

[btag508-B40] Zhao J , Min W. SpaICL: image-guided curriculum strategy-based graph contrastive learning for spatial transcriptomics clustering. Brief Bioinform 2025;26:bbaf433. 40838787 10.1093/bib/bbaf433 PMC12368861

[btag508-B41] Zhen P , Wang X, Shu H et al Disconst: distribution-aware contrastive learning for spatial domain identification. Genomics Proteomics Bioinform 2025;24:qzaf085. 10.1093/gpbjnl/qzaf085 PMC13317986 40990806

[btag508-B42] Zhu P , Shu H, Wang Y et al MAEST: accurately spatial domain detection in spatial transcriptomics with graph masked autoencoder. Brief Bioinform 2025;26:bbaf086. 40052440 10.1093/bib/bbaf086 PMC11886571

