# Supplementary material for: Structural-information guided fusion for spatial domain identification from spatial transcriptomics
Source: Bioinformatics. 2026 Jul 9;42(7):btag508. doi: 10.1093/bioinformatics/btag508 (PMC13395104; doi:10.1093/bioinformatics/btag508)
Supplement: btag508_Supplementary_Data [file btag508_supplementary_data.docx]

Supplementary Materials for

**Structural information-guided fusion for spatial domain identification from spatial transcriptomics**

Min Zhang,^1,2^ Xiaoke Ma,^1,2,3∗^ Cheng Chen^4^, Xin Chen,^5∗^ Peng Gao^6^ and Shaoqing Feng^7∗^

^1^ School of Computer Science and Technology, Xidian University, No.2 South Taibai Road, Xi'an, 710071, Shaanxi, China.

^2^ Key Laboratory of Smart Human-Computer Interaction and Wearable Technology of Shaanxi Province, Xidian University, No.2 South Taibai Road, Xi'an, 710071, Shaanxi, China.

^3^ Shenzhen Loop Area Institute, No. 6 Hongmian Road, Fubao District, Shenzhen, 518017, Guangdong, China.

^4^ MOE Key Laboratory of Bioinformatics, BNRIST Bioinformatics Division, Institute for Precision Medicine & Department of Automation, Tsinghua University, Beijing 100084, China;

^5^ Department of Radiology, School of Medicine, The Second Affiliated Hospital of South China University of Technology (Guangzhou First People’sHospital), Guangzhou, 510180, China.

^6^ Department of Hematology, The First Affiliated Hospital of Xian jiaotong University, No. 277 Yanta West Road, 710061, Xi’an Shaanxi, China.

^7^ Department of Plastic and Reconstructive Surgery, Shanghai Ninth Peoples Hospital, Shanghai Jiaotong University, No. 639 Zhizaoju Road, 200011,Shanghai, China.

Corresponding authors. Xiaoke Ma, E-mail:xkma@xidian.edu.cn; Shaoqing Feng, E-mail:fengsq112046@sh9hospital.org.cn; Xin Chen,mail:wolfchenxin@163.com.

**Keywords:** Spatial domain, Pathway activity, Spatially resolved transcriptomics, Graph fusion

1. **Supplementary Figures**

Table S1. Ablation study on the DLPFC and osmFISH datasets

| $\text{L}_{\text{ZINB}}$ | $\text{L}_{\text{DSI}}$ | $\text{L}_{\text{SBPR}}$ | DLPFC(151672) | osmFISH |
| --- | --- | --- | --- | --- |
|  |  | √ | 0.108 | 0.042 |
|  | √ |  | 0.598 | 0.628 |
|  | √ | √ | 0.599 | 0.629 |
| √ |  |  | 0.228 | 0.382 |
| √ |  | √ | 0.449 | 0.434 |
| √ | √ |  | 0.628 | 0.682 |
| √ | √ | √ | 0.861 | 0.751 |

**
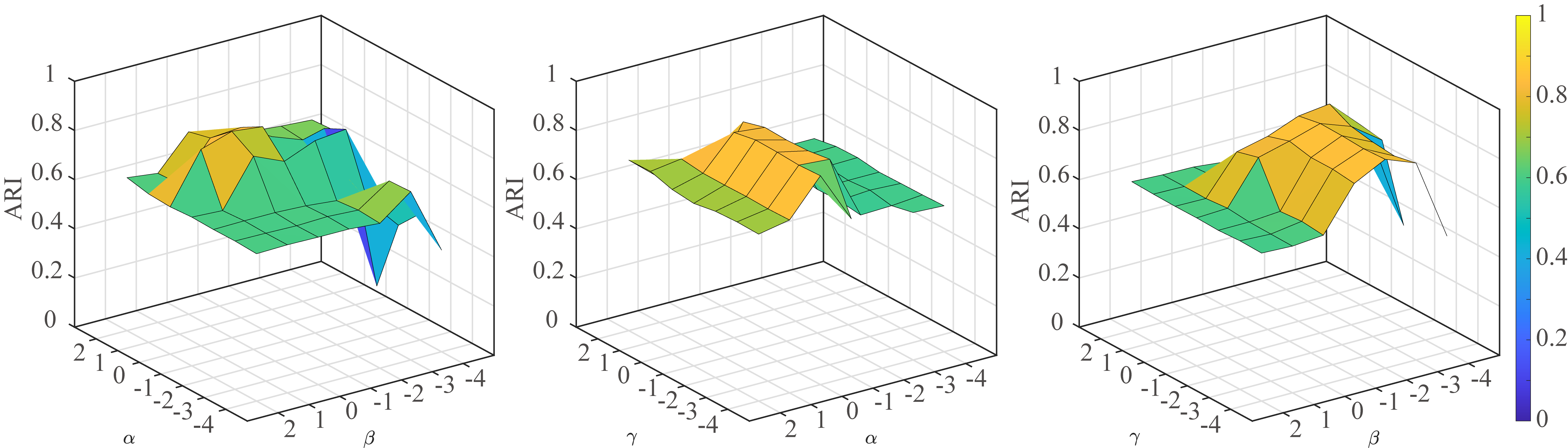
**

**Fig. S1** Parameter analysis of SGFST on 151672 slices of DLPFC


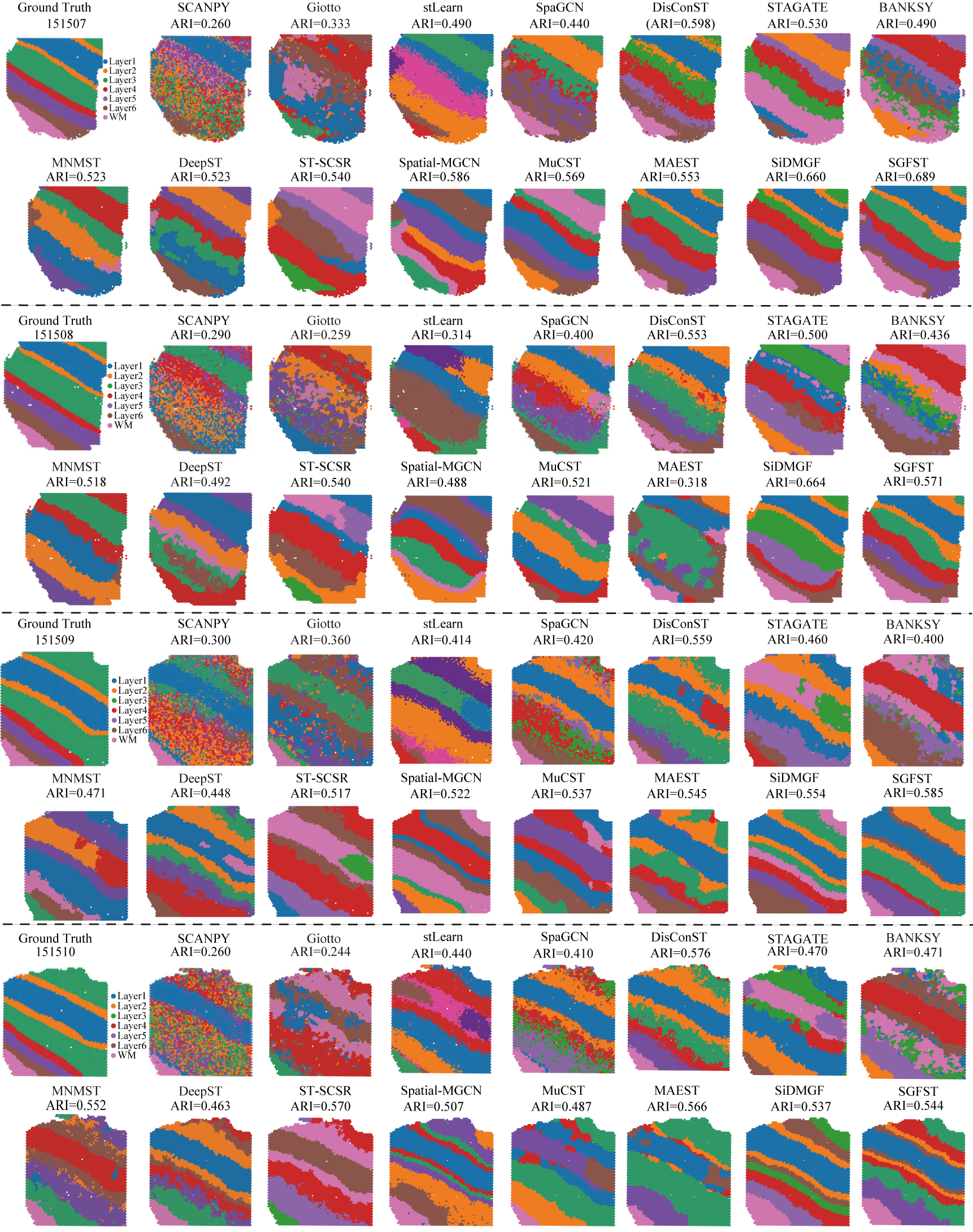


**Fig. S2** Performance of various algorithms for spatial domain identification on Annotated dorsolateral prefrontal cortex (DLPFC, http://spatial.libd.org/spatialLIBD) data (151507, 151508, 151509, 151510), where ground truth spots are mapped on their spatial location, divided into various cortical layers (L1-L6 or L3-L6) and white matter (WM) layer.


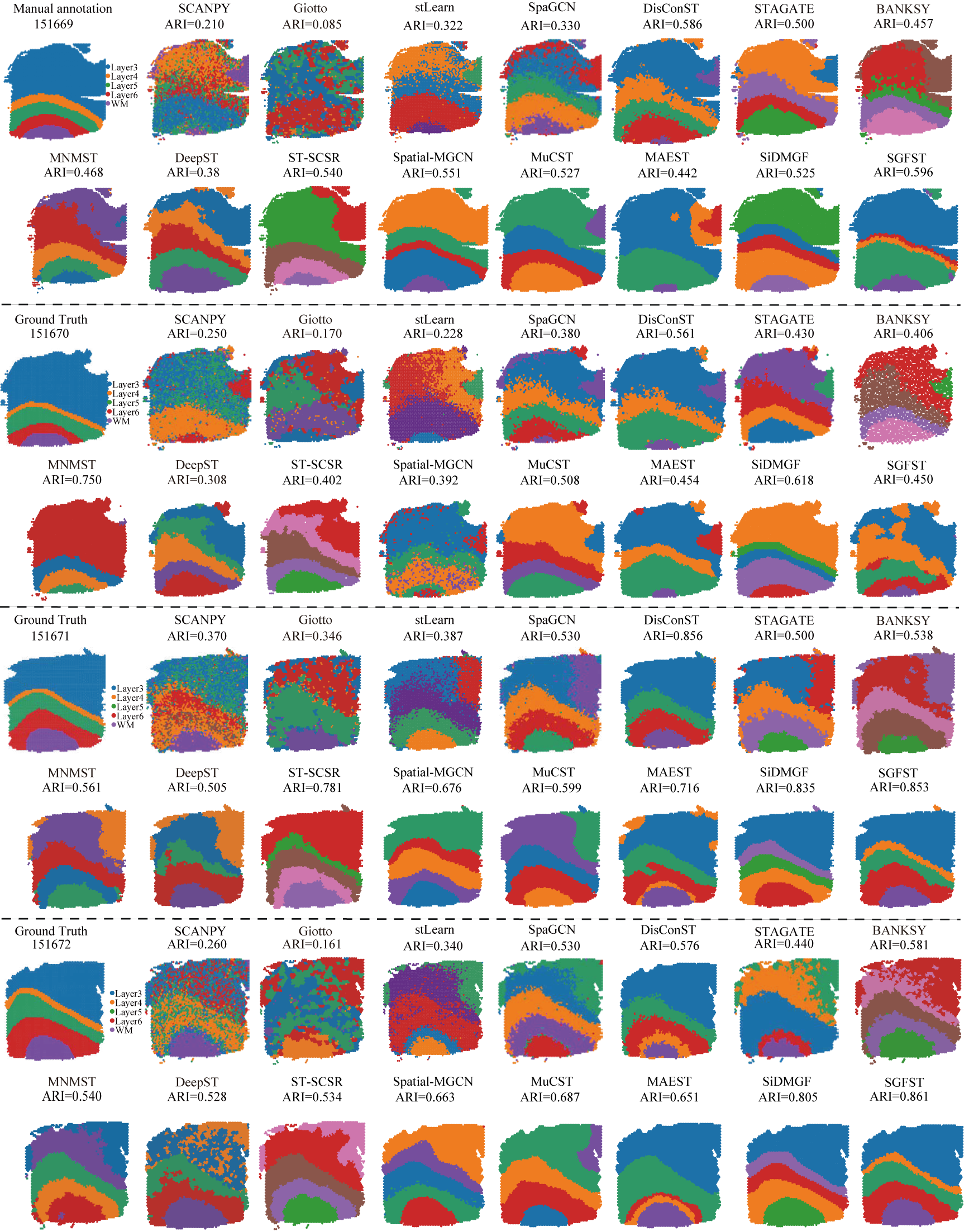


**Fig. S3** Performance of various algorithms for spatial domain identification on Annotated dorsolateral prefrontal cortex (DLPFC, http://spatial.libd.org/spatialLIBD) data (151669, 151670, 151671, 151672), where ground truth spots are mapped on their spatial location, divided into various cortical layers (L1-L6 or L3-L6) and white matter (WM) layer.


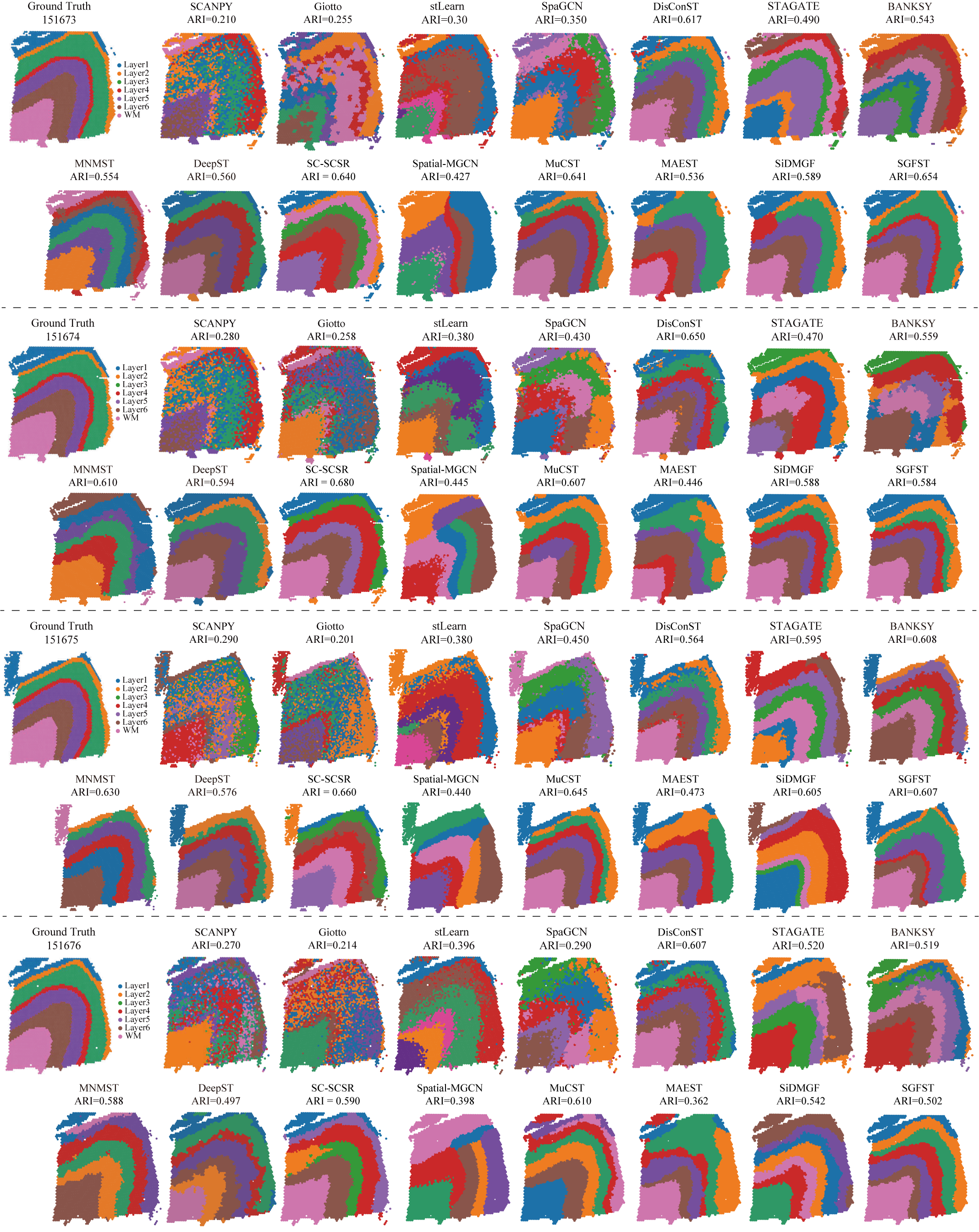


**Fig. S4** Performance of various algorithms for spatial domain identification on Annotated dorsolateral prefrontal cortex (DLPFC, http://spatial.libd.org/spatialLIBD) data (151673, 151674, 151675, 151676), where ground truth spots are mapped on their spatial location, divided into various cortical layers (L1-L6 or L3-L6) and white matter (WM) layer.


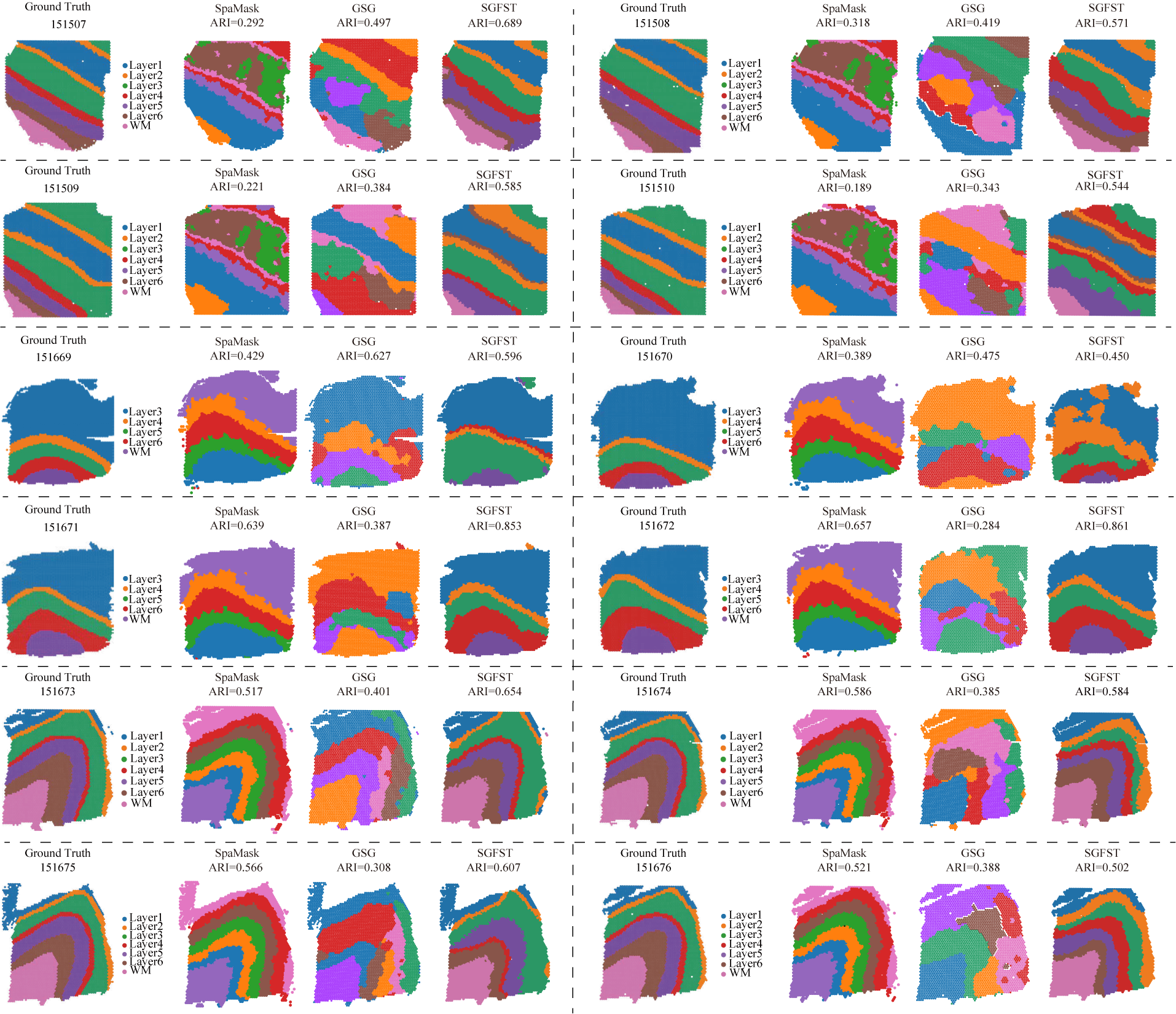


**Fig. S5** Performance of SpaMask, GSG and SGFST for spatial domain identification on Annotated dorsolateral prefrontal cortex (DLPFC, http://spatial.libd.org/spatialLIBD) data, where ground truth spots are mapped on their spatial location, divided into various cortical layers (L1-L6 or L3-L6) and white matter (WM) layer.


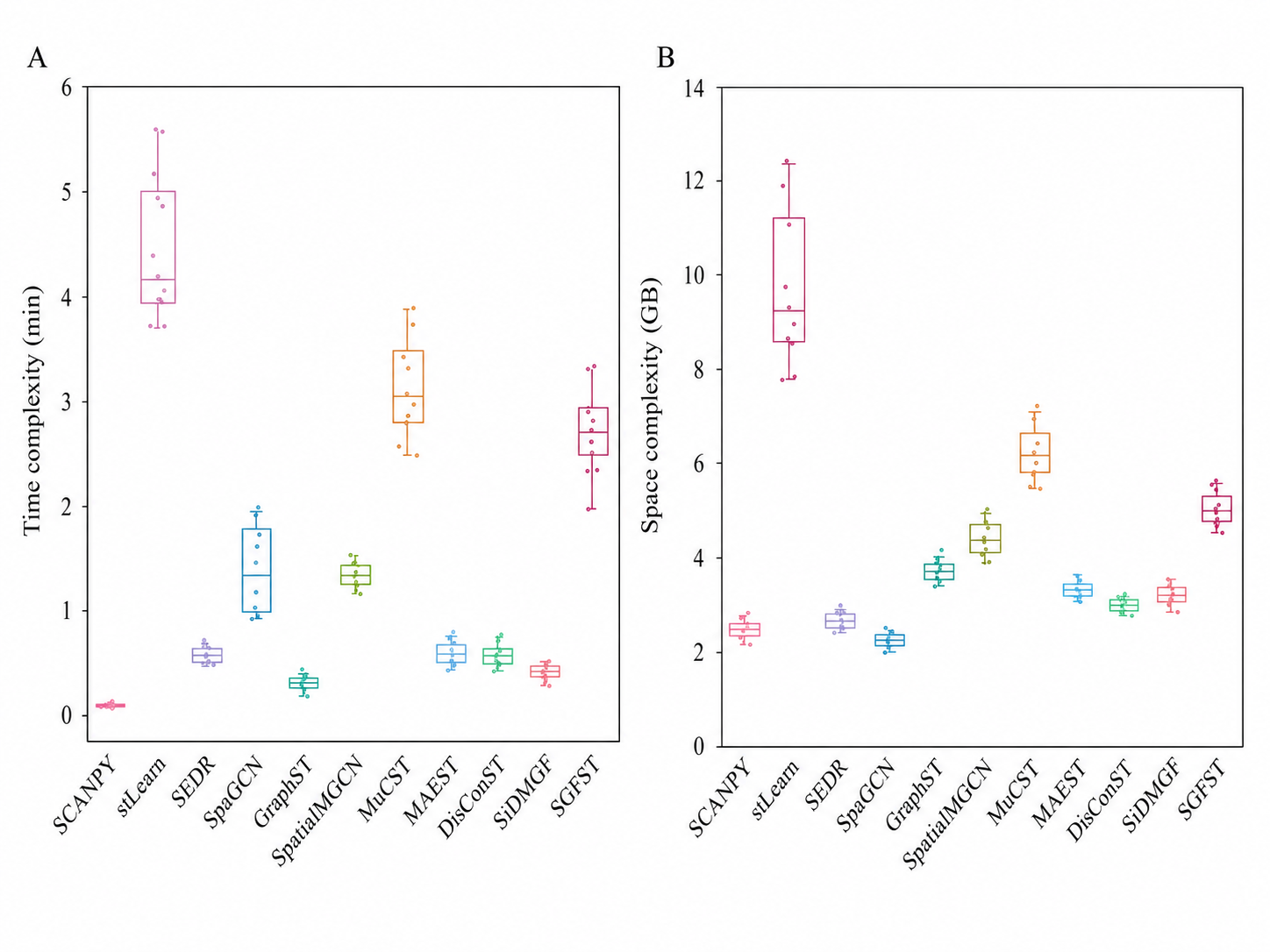


**Fig. S6** Boxplot of running time **(A)** and memory usage **(B)** of different algorithms on all slices of DLPFC dataset, where the center line, box limits and whiskers denote the median, upper and lower quartiles and 1.5$\times$ interquartile range, respectively.


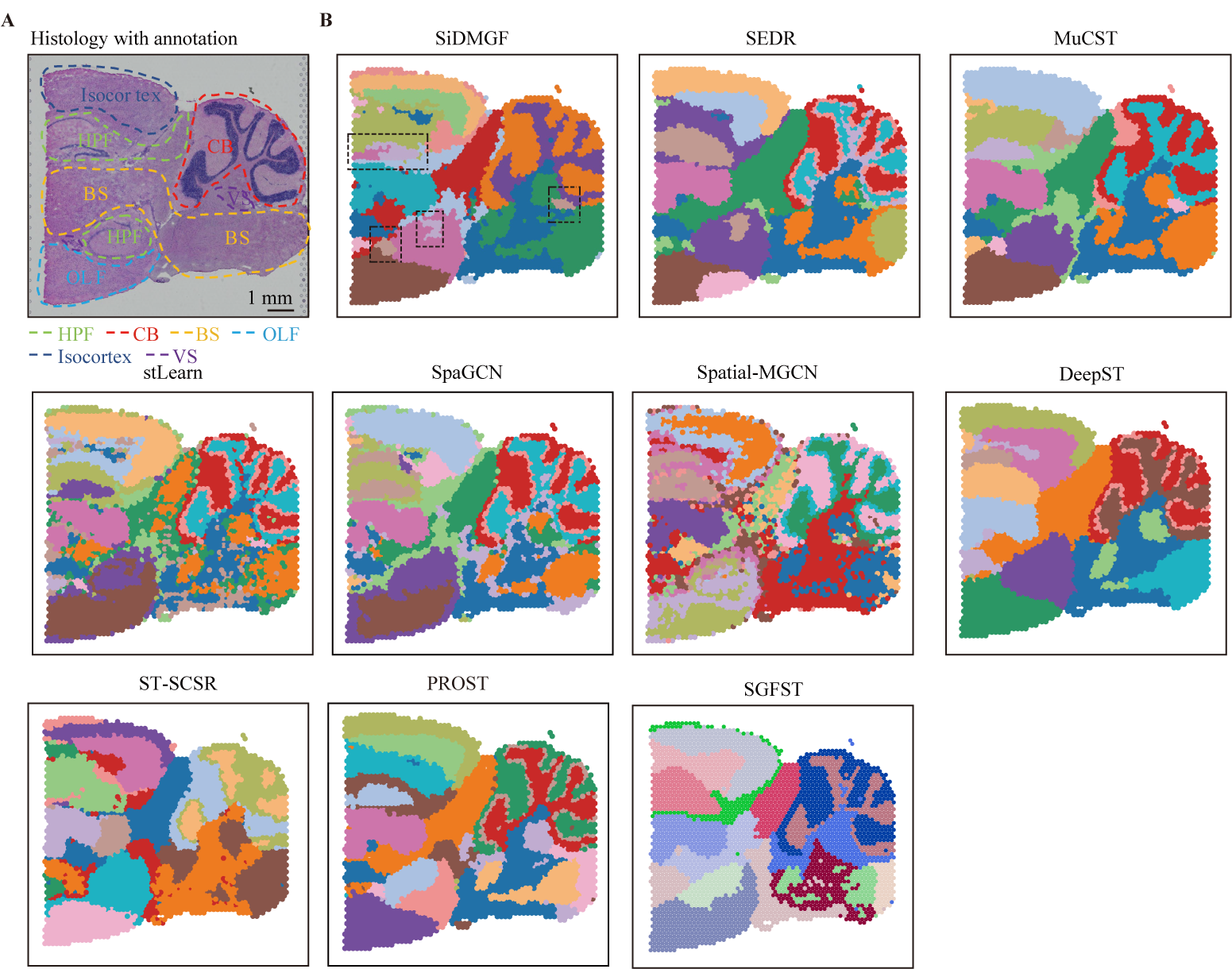


**Fig. S7** SGFST identifies complex spatial domain structures in mouse posterior brain data. (A) Annotated histology image of mouse posterior brain slice. (B) Spatial domains identified by different methods, illustrating their ability to delineate anatomical structures in the posterior brain, where the black dashed lines highlight a narrow region that is correctly delineated by SGFST but not captured by most baseline methods


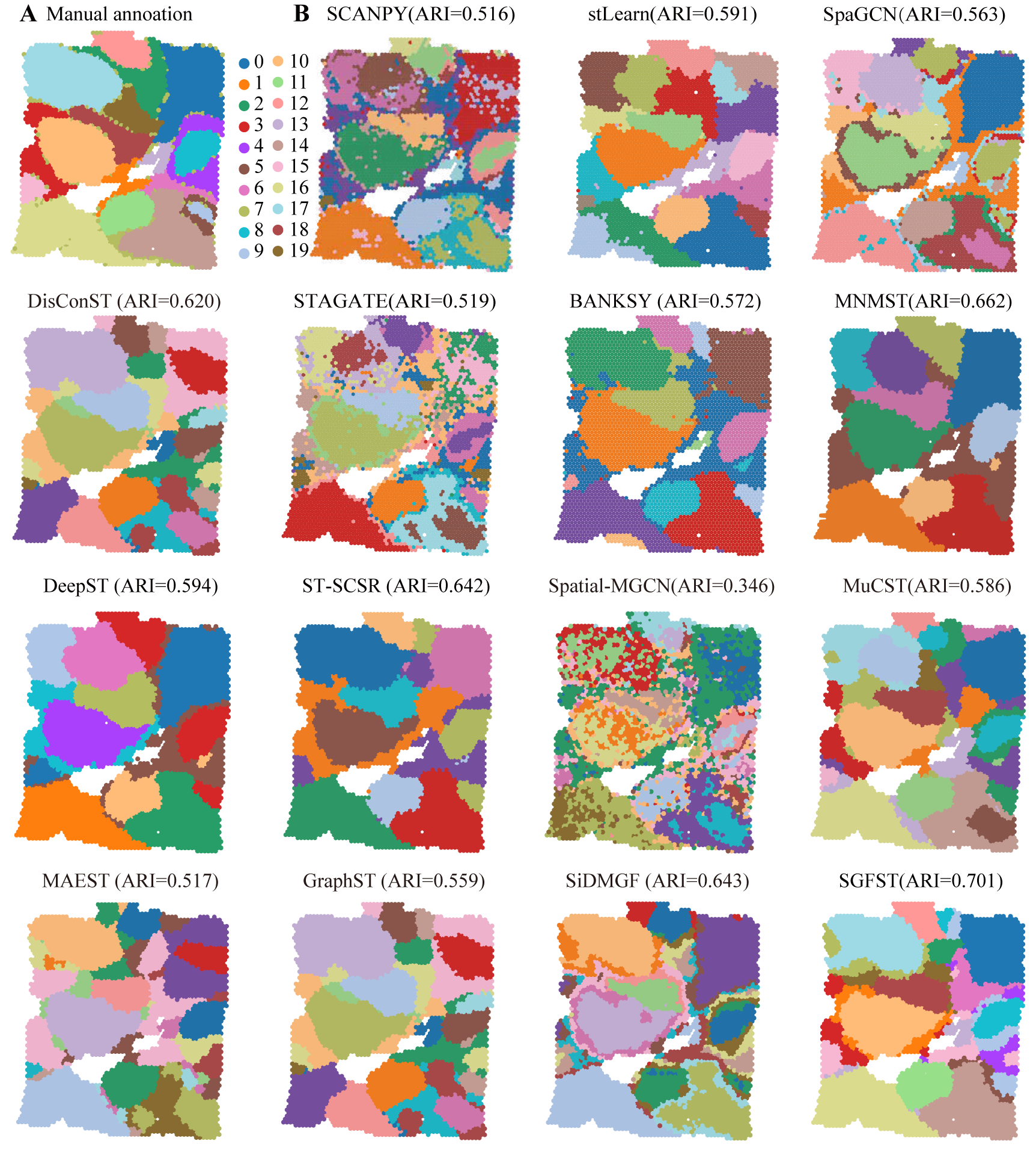


**Fig. S8** Comparison of spatial domains identified by SCANPY, stLearn, SpaGCN, GraphST, DisConST， STAGATE, BANKSY, MNMST, DeepST, ST-SCSR, Spatial-MGCN, MuCST, MAEST,GraphST, SiDMGF and SGFST on the human breast cancer data, and visualization of identified spatial domains (domains=20), where spots are colored by the identified spatial domains.


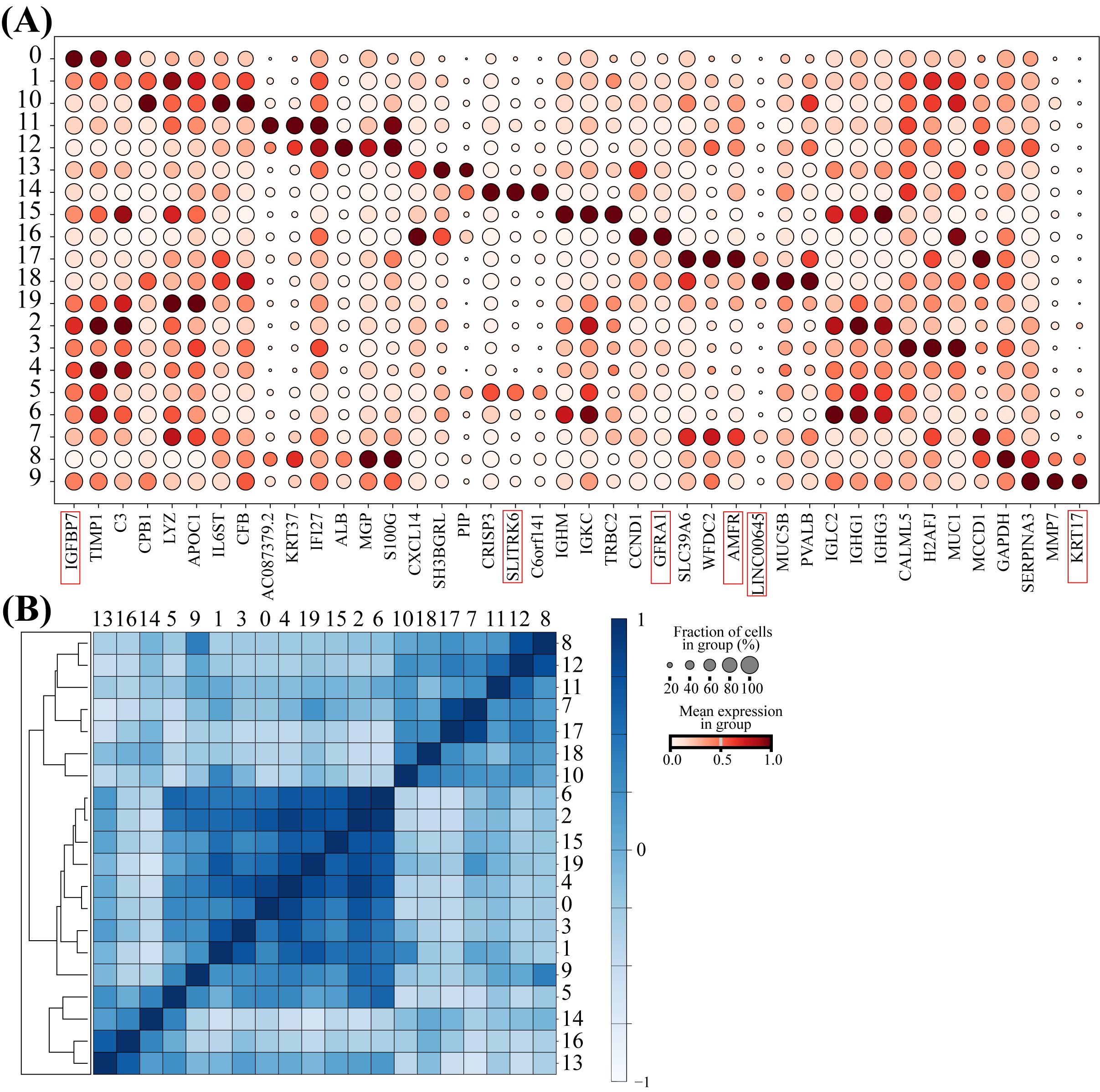


**Fig. S9** (A) Domain-specific expression patterns of representative marker genes shown by dot plot. (B) Heatmap of Pearson correlation coefficients among identified domains. Morphotype annotations on the left are obtained by mapping the identified domains to the manual annotations.


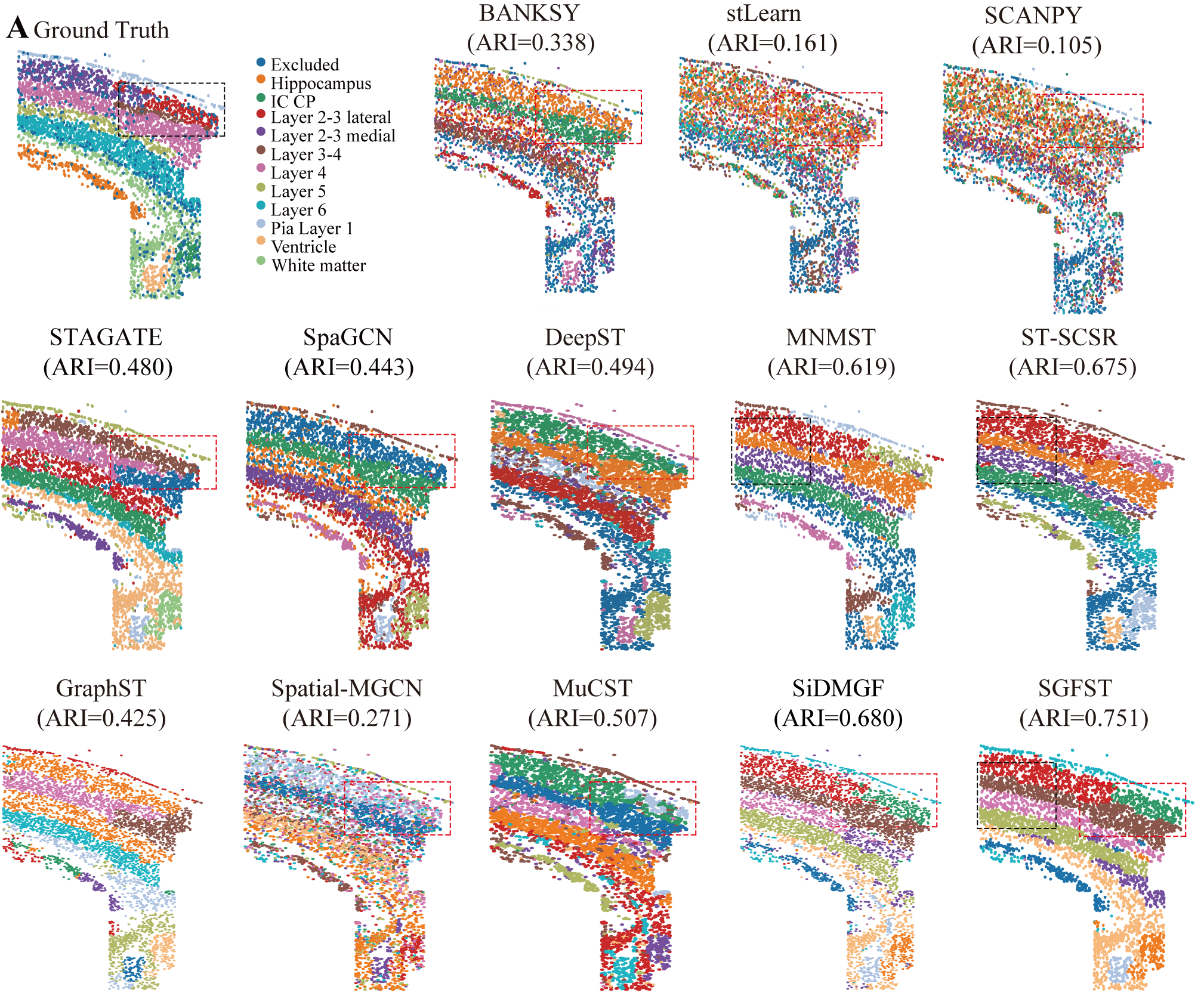


**Fig. S10** Visualization of spatial domains identified by different algorithms on mouse cortex slice sequenced by osmFISH, where spots are colored by the identified spatial domains.


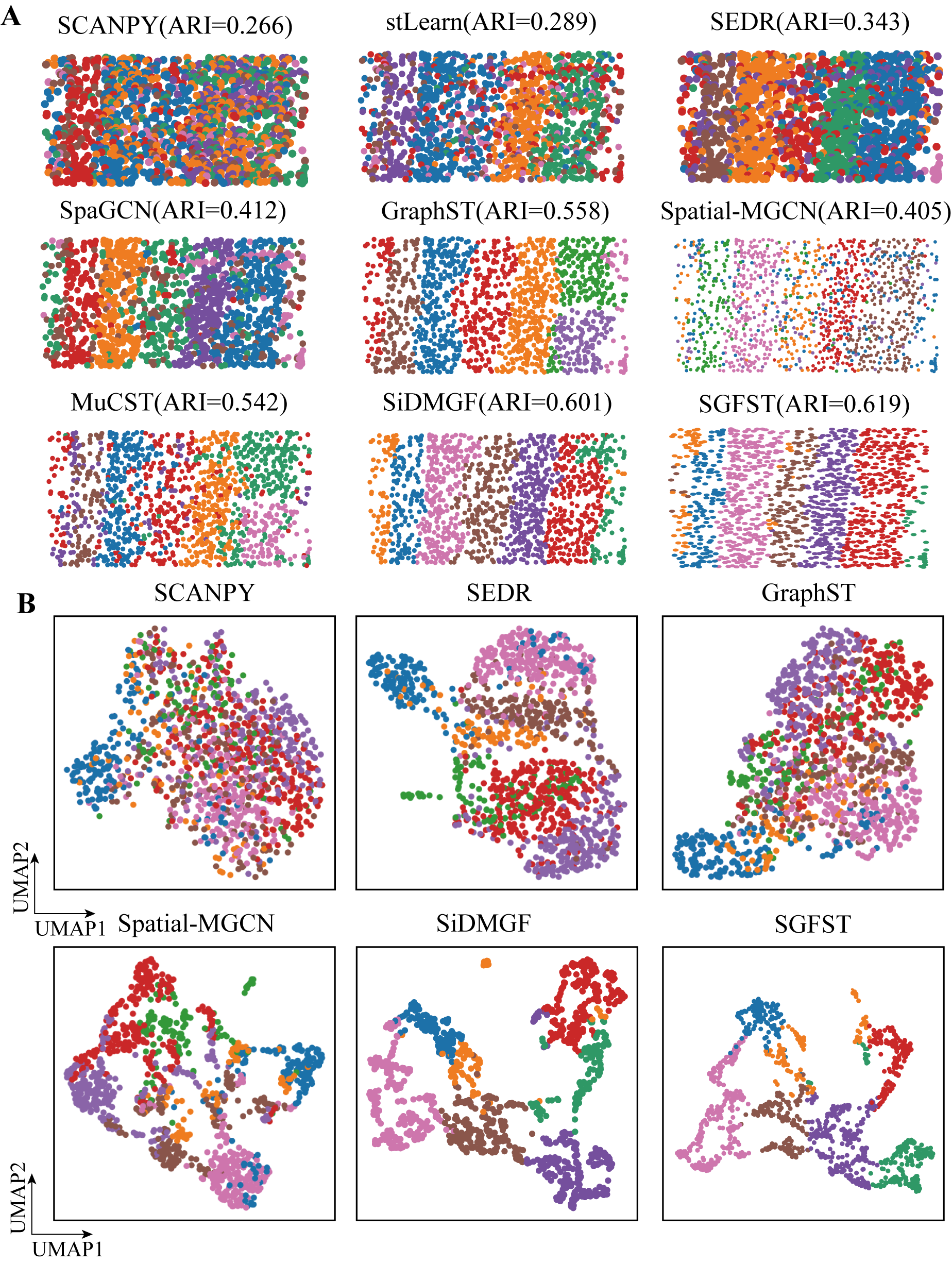


**Fig. S11** **(A)** Visualization of spatial domains identified by SCANPY, stLearn, SEDR, SpaGCN, GraphST, Spatial-MGCN, MuCST, SiDMGF and SGFST on STARmap data. **(B)** UMAP plots of cell embeddings learned by SCANPY, SEDR, GraphST, Spatial-MGCN, SiDMGF and SGFST where spots are colored by the identified spatial domains.


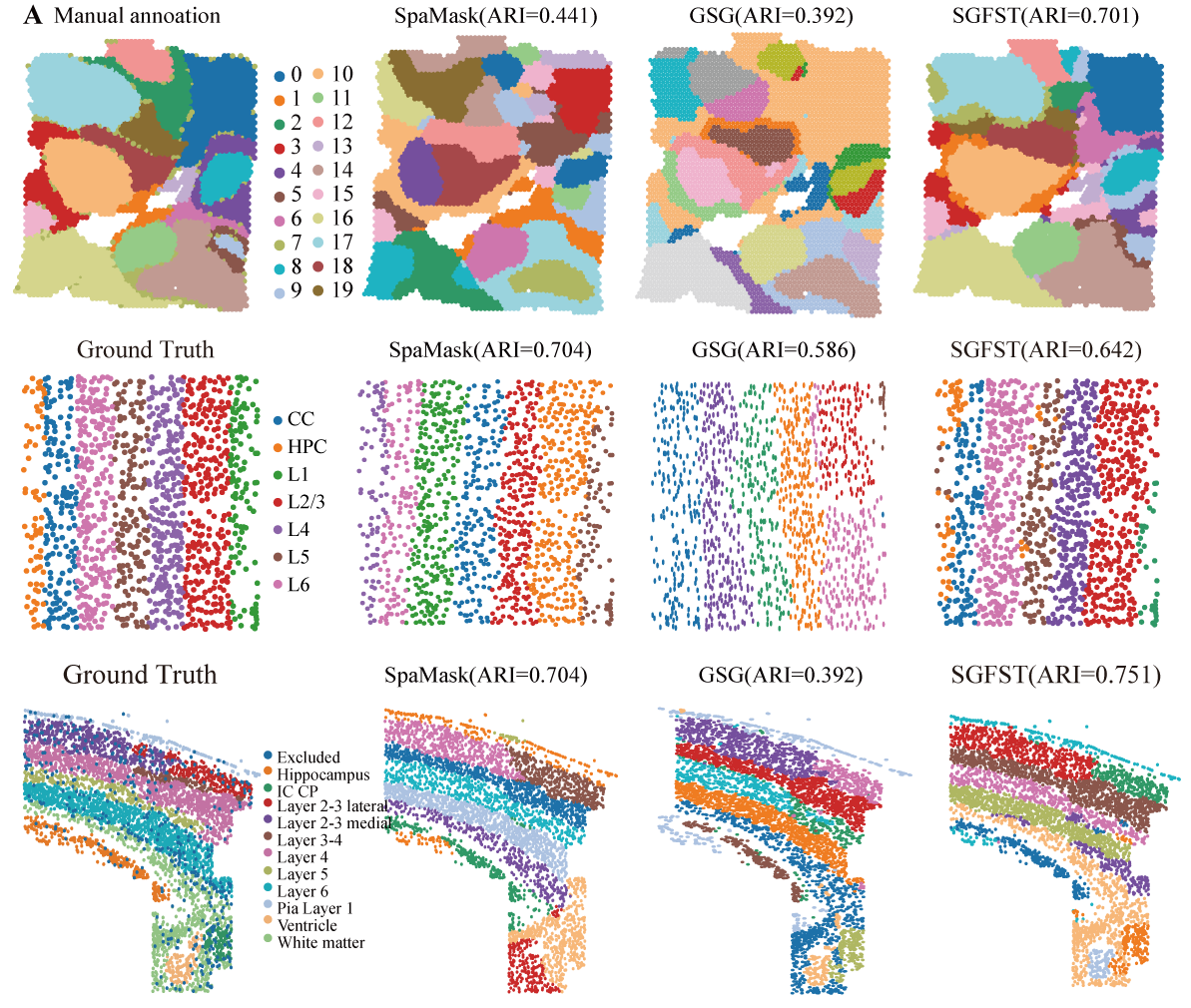


**Fig. S12** Visualization of spatial domains identified by different algorithms on breast cancer, STARmap, osmFISH.
